# Establishing Computational Approaches Towards Identifying Malarial Allosteric Modulators: A Case Study of *Plasmodium falciparum* Hsp70s

**DOI:** 10.3390/ijms20225574

**Published:** 2019-11-08

**Authors:** Arnold Amusengeri, Lindy Astl, Kevin Lobb, Gennady M. Verkhivker, Özlem Tastan Bishop

**Affiliations:** 1 Research Unit in Bioinformatics (RUBi), Department of Biochemistry and Microbiology, Rhodes University, Grahamstown 6140, South Africa; ahnoamu@gmail.com (A.A.); K.Lobb@ru.ac.za (K.L.); 2 Graduate Program in Computational and Data Sciences, Schmid College of Science and Technology, Chapman University, Orange, CA 92866, USA; astl100@mail.chapman.edu (L.A.); verkhivk@chapman.edu (G.M.V.); 3 Department of Chemistry, Rhodes University, Grahamstown 6140, South Africa; 4 Department of Biomedical and Pharmaceutical Sciences, Chapman University School of Pharmacy, Irvine, CA 92618, USA; 5 Department of Pharmacology, Skaggs School of Pharmacy and Pharmaceutical Sciences, University of California San Diego, 9500 Gilman Drive, La Jolla, CA 92093, USA

**Keywords:** heat shock proteins, South African natural compounds, allosteric drugs, dynamic residue networks, betweenness centrality

## Abstract

Combating malaria is almost a never-ending battle, as Plasmodium parasites develop resistance to the drugs used against them, as observed recently in artemisinin-based combination therapies. The main concern now is if the resistant parasite strains spread from Southeast Asia to Africa, the continent hosting most malaria cases. To prevent catastrophic results, we need to find non-conventional approaches. Allosteric drug targeting sites and modulators might be a new hope for malarial treatments. Heat shock proteins (HSPs) are potential malarial drug targets and have complex allosteric control mechanisms. Yet, studies on designing allosteric modulators against them are limited. Here, we identified allosteric modulators (SANC190 and SANC651) against *P. falciparum* Hsp70-1 and Hsp70-x, affecting the conformational dynamics of the proteins, delicately balanced by the endogenous ligands. Previously, we established a pipeline to identify allosteric sites and modulators. This study also further investigated alternative approaches to speed up the process by comparing all atom molecular dynamics simulations and dynamic residue network analysis with the coarse-grained (CG) versions of the calculations. Betweenness centrality (BC) profiles for PfHsp70-1 and PfHsp70-x derived from CG simulations not only revealed similar trends but also pointed to the same functional regions and specific residues corresponding to BC profile peaks.

## 1. Introduction

Malaria, caused by parasites of the genus *Plasmodium* and transmitted via Anopheles mosquitoes, remains a major public health problem mostly in Asia, Africa and South America [1]. Despite the large number of antimalarial drugs developed so far, the recurrent ability of the parasite to gain resistance against them is a serious concern. This concern was strongly raised again due to the recent emergence of resistance in *Plasmodium falciparum* in Southeast Asia to currently used artemisinin-based combination therapies (ACTs) [2]. Although ACTs have provided recognizable levels of reduction in malaria cases, if the resistant parasite strains spread to Africa, the scenario might lead to catastrophic consequences. To prevent such results, we need to start thinking out of the box, and find non-conventional approaches to identify effective drug targets, drug targeting sites and new drugs. Traditionally, computer aided drug design includes structure based and ligand based approaches, and mostly targets the active sites [3,4,5,6]. In this study we propose to look at allosteric hot spots of selected *P. falciparum* 70kDa heat shock proteins (PfHsp70s) and to identify allosteric modulators against them. Allosteric drugs might be our new hope for malarial treatments as they have many potential advantages over orthosteric drugs. Allosteric drugs are highly selective, hence less toxic. Even though heat shock proteins are potentially good drug targets against cancer and infectious diseases as indicated in a number of studies [7,8,9], it is a known fact that inhibitors designed against them show high toxicity [7,8]. 

The Hsp70s, highly conserved molecular chaperones, play an important role in maintaining cellular proteostasis [10]. They are made up of two coordinated functional domains: A substrate binding domain (SBD) and a nucleotide binding domain (NBD), connected via a conserved interdomain linker. During their functional cycle, Hsp70s undergo major conformational changes, which are regulated through interdomain allosteric communication in relation to the bound states of the NBD and SBD [11,12]. The protein allosterically alternates between a closed (substrate peptide and ADP bound) and an open (ATP bound, substrate-free) conformation [13,14]. The open conformation adopts a compact spatial arrangement: The NBD and SBD are coupled via the interdomain linker forming a NBD-SBD interface [14,15]. The closed conformation occupies an elongated spatial arrangement: The NBD and SBD operate as individual units exposing the linker to solvent [13,16]. ATP binding promotes domain docking, A-LID dissociation from the β-SBD, and substrate release activities [13,14,17]. The protein transition to the open state characterized by low substrate affinity and high on/off substrate kinetics [13,17]. Substrate rebinding enhances ATP hydrolysis, promotes domain dissociation, and closure of the A-LID trapping the bound substrate [13,18]. The protein transition to the closed state is characterized by high substrate affinity, and low exchange rates [13,17]. 

In general, *P. falciparum* heat shock proteins present potential targets for antimalarial vaccine and drug design [19]. *P. falciparum* encodes six main isoforms of Hsp70s [20,21]: PfHsp70-x, PfHsp70-y, PfHsp70-z, PfHsp70-1, PfHsp70-2, and PfHsp70-3. Of interest in this study are PfHsp70-x and PfHsp70-1. PfHsp70-1 is expressed throughout the clinical stages of the parasites’ life cycle [22], whereas PfHsp70-x is maximally expressed at the schizont stage [23]. Effectively, PfHsp70-x and PfHsp70-1 are expressed during the critical asexual blood developmental stage of *P. falciparum* [23,24]. Moreover, both proteins are well recognized by antibodies in asymptomatic infections [25]. PfHsp70-1, localized in the nucleus and cytosol [20], is essential for parasitic survival [26]. The protein possesses a C-terminal EEVD regulatory motif implicated in recognition and binding of other chaperones [27,28]. PfHsp70-1 is known to work co-operatively with other chaperones and co-chaperone, including PfHsp90 and DnaJ [29,30]. Up regulation of PfHsp70-1 due to radical shifts in environmental conditions during the parasite’s life cycle is thought to improve stress endurance hence survival prospects [31,32]. For instance, overexpression of Hsp70’s in high dose artesunate environment [32,33] has been implicated in emergence of drug-resistant *P. falciparum* strains in Southeast Asia [2]. This implies that the inhibition of PfHsp70-1 could not only generate a cascade of inhibitory effects downstream leading to fatal consequences, but also arrest the parasites’ coping maneuvers.

Unlike PfHsp70-1, PfHsp70-x possesses an EEVN C-terminal peptide thought to modulate its recognition to host co-chaperones [34]. Additionally, the protein possesses an extended N-terminal suggested to be an endoplasmic reticulum (ER) signaling peptide [20,23]. PfHsp70-x is unique to *P. falciparum* and is the only 70kDa heat shock protein of parasitic origin shown to localize to the erythrocyte cytosol [23,35]. This property hints at monopolous involvement of PfHsp70-x in regulatory processes beyond intraparasitic confines. It is likely that PfHsp70-x accompanies exported proteins to various host cell compartments, effectively maintaining their functional integrity throughout. Despite recent reports showing that the deletion of PfHsp70-x bears inconsiderable effects on *P. falciparum* survival in vitro [35,36], its knockout resulted in less efficient export of *Plasmodium falciparum* erythrocyte membrane protein 1 (PfEMP1), a key virulence factor, leading to weak cytoadherence characteristics [35]. These findings provided the crucial evidence of PfHsp70-x’s role in leveraging parasite-induced virulence, hence its importance in pathogenicity.

Even though aspects of their complex allosteric control mechanism have been thoroughly investigated via wet-lab studies as well as computational approaches [11,14,37,38], most of the effort in drug discovery went to inhibitor design against NBD [7,39,40]. To date, there are only few attempts to identify allosteric modulators against Hsps in general [41,42,43,44]. In our previous studies, we used all-atom molecular dynamics (MD) simulations coupled with perturbation response scanning (PRS) to determine allosteric hotspots that may be implicated in modulating conformational dynamics in *E. coli* Hsp70 and DnaK [11] as well as human Hsp90 [45]. We then proposed that force perturbations at these hotspot sites might be a natural consequence of binding forces caused by protein–protein or protein–ligand interactions; and that these sites may be suitable allosteric drug target candidates. This hypothesis was, then, successfully applied to identification of allosteric modulators against human Hsp90 protein [42] and human Hsp72 & Hsc70 [43] proteins as cancer drug targets.

In this study, we used our well-established pipeline to further identify allosteric modulators against PfHsp70-x and PfHsp70-1. We hypothesize that the binding of novel compounds to allosteric sites may modulate the allosteric transition dynamics of PfHsp70-x/PfHsp70-1 leading to enhanced/reduced modes of action. One hundred nanosecond all-atom MD simulations revealed SANC190 and SANC651 as promising PfHsp70-x and PfHsp70-1 modulators. SANC190 and SANC651 binding to PfHsp70-x triggered structural rearrangements that favored domain advances. Dynamic residue network analysis revealed several transformed residues corresponding to critical allosteric communication hubs previously implicated in ATP induced closed to open conformation transition in *E. coli* Hsp70. Our findings suggested that SANC190 and SANC651 could engender allosteric agonism promoting NBD-SBD coupling and further accelerating ATP induced domain docking kinetics. The results support the strategy of modulating communication networks of allostery controlled proteins for the development of next generation antimalarial agents. We also investigated alternative approaches towards identifying allosteric modulators to hasten the process by comparing all atom MD simulations and dynamic residue network (DRN) analysis with the coarse-grained (CG) version of the calculations. CG calculations showed similar trends with all atom MD and DRN results.

## 2. Results and Discussion

### 2.1. Phylogenetic Tree Analysis

**Figure 1:**
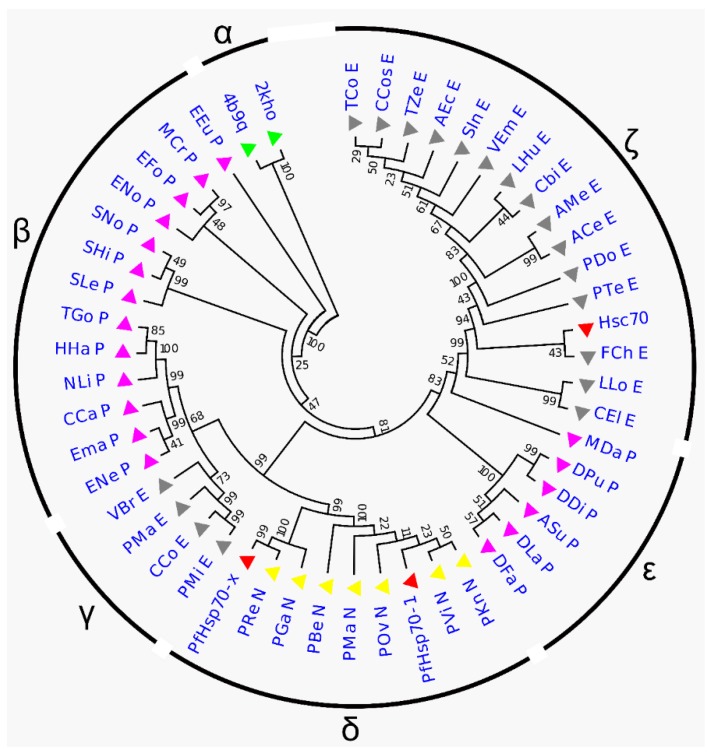
Maximum likelihood phylogenetic tree based on Hsp70 sequences showing the relationship between host (Hsc70) and parasitic proteins (PfHsp70-x and PfHsp70-1). Color code: Red: PfHsp70-x, PfHsp70-1, and Hsc70; purple: 7 non-falciparum (plasmodial); grey: 19 Protozoan (non-plasmodial); blue: 19 Eukaryotic (non-protozoan); yellow: 2KHO and 4B9Q. Percentage recurrence of 1000 bootstrap tests is indicated next to the branches.

To estimate the distance between parasitic (PfHsp70-x, PfHsp70-1) and human (Hsc70) Hsp70 lineages, a phylogenetic tree was constructed from a dataset of 50 homologous Hsp70 sequences (Table S1). Generally, protein sequences were clustered into six groups: α, β, γ, δ, ε, and ζ (Figure 1, Figure S1). Outgroup sequences (2KHO and 4B9Q) (group α) branched early with 100% partition frequency, indicating that the results were dependable. PfHsp70-x and PfHsp70-1 were grouped within the plasmodium cluster (group δ) as expected, suggesting that the proteins are closely related. Notably, PfHsp70-x produced a distinct clade with *P. reichenowi*, and *P. gaboni* recording a 99% partition frequency. Also, the triad were confirmed to possess the characteristic extended N-terminal signal peptide segment suggesting a common descent [20,46]. Besides the three, all sequences of plasmodial origin, including PfHsp70-1, formed a nested clade. Hsc70 was grouped within the eukaryotic cluster (group ζ), signifying that the human protein is distantly related to *P. falciparum* proteins. The above results suggest that parasitic and host sequences share distinguishable ancestral origin and thus, could be selectively modulated.

### 2.2. Homology Modeling

Efforts to understand binding specificities of potential *P. falciparum* Hsp70 modulators have often been frustrated by absence of 3D structural information from experimental data. We obtained 3D coordinates of target proteins PfHsp70-x, PfHsp70-1, and Hsc70 by implementing comparative modeling technique. PRIMO [47], a web-based tool for homology modeling, incorporates robust search and comparison algorithms, including protein BLAST [48] and HHsearch [49] for faster template recognition and optimal alignment. Objectively, full-length structures solved in both open and closed conformation were considered. Often, sequences that share 30% or more pairwise identity possess comparable structures [50]. Nearly full-length close homologs PDB ID: 2KHO and 4B9Q_chain A properly aligned to 88% and 85% (on average) of each query sequence, registered a sequence identity score of 49% and 50% (on average) against each query sequence respectively, and possessed fairly high resolution geometries (Table S2A). Both templates and targets were incorporated in a multiple sequence alignment with 45 homologous Hsp70 sequences to ensure conserved patterns were retained and thus improve the alignment accuracy. Alignment profiles for each protein conformation were generated and models were built using MODELLER version 9.12 [51]. Due to the large number of models produced, initial global quality assessment using MODELLER’s z-DOPE score [52] was performed and models ranked. Fundamentally, structures of desirable quality should register a value of −0.5 or below [52]. All top models recorded z-DOPE scores below −0.51 indicating native-like representations (Table S2B). PROCHECK Ramachandran plot [53] provides a detailed and comprehensive description of the distribution of amino-acid dihedrals (φ and ψ). All models recorded at least 92.90% of residues in sterically allowed regions (favored regions) (Table S2B) indicating that the structures built possessed desirable stereochemical qualities. Evaluation of the quality of structure (3D) to sequence (1D) match using VERIFY3D [54] exhibited satisfactory results suggesting reliability of the models; at least 83.99% of amino acids across all models registered scores ≥0.2 in the 3D/1D profile.

### 2.3. Molecular Docking

High-throughput virtual screening was employed to screen parasitic (PfHsp70-x, PfHsp70-1) and human (Hsc70) proteins against a library of 623 natural compounds for potential parasite-specific inhibitory activity. Docking programs AutoDock Vina [55] followed by AutoDock4 [56] were used. To validate the docking programs, co-crystallized ADP and ATP were redocked into 3ATV (open conformation) and 4B9Q (closed conformation) proteins respectively. The RMSD values of 0.26 Å (ADP) and 0.79 Å (ATP) for AutoDock Vina, and 0.54 Å (ADP) for AutoDock4 between co-crystallized and docked ligand poses indicated the reliability of both tools and docking parameters applied to reproduce experimental poses. 

Initially, AutoDock Vina was used to screen each protein conformation. Ten docked poses were generated for each docking trial and ranked using Vina scoring function. The top ranked pose was selected for post-docking analysis. Binding energy heatmaps of all proteins versus all ligands (Figure S2A(i),B(i)) were plotted to get an overview of how well the library was performing against each protein conformation. These plots revealed that the open conformation of PfHsp70-x, and specifically the substrate binding domain (SBD) was more receptive to small molecules, thus a promising target protein and domain. Based on binding energy difference heatmaps between parasitic (PfHsp70-x and PfHsp70-1) and human (Hsc70) proteins (Figure S2A(ii),B(ii)), it was more evident that the SBD of both PfHsp70-x and PfHsp70-1 (open conformations) possessed superior binding affinities to ligands than Hsc70, and would therefore formulate suitable targets for selective drug design. Docked ligand poses in PfHsp70-x SBD were visually inspected using PyMOL [57]. We observed that docked ligands mainly clustered in two non-native ligand binding sites: β-SBD back pocket and β-SBD–A-LID interface (Figure S2C). The β-SBD back pocket is thought to be an allosteric ligand-binding site [11,58] and was targeted in human Hsp72 and Hsc70 for development of anticancer modulators in our previous study [58]. The β-SBD–A-LID interface overlaps critical residues stabilizing the shut lid [59], besides various residues participating in substrate binding activities [11], suggesting a novel allosteric ligand-binding site. Considering its proximity to the bound substrate, it is likely that any bound ligand could unbalance protein-substrate thermodynamics. Focusing on PfHsp70-x open conformation, ligands docked to the two sites above were isolated using in house Python scripts for further analysis. 

Typically, drug discovery programs employ high throughput screening paradigms with the intent to identify high affinity binders to target macromolecules [60,61]. Despite the traditional practice, selection of the most favorable solution remains an arduous task [61,62]. Identification of selective allosteric modulators, especially against evolutionary conserved targets presents per se a complex endeavor. Here, we utilized two properties characteristic of ligand binding sites [61] to gain selectivity: (I) Shape complementarity to the ligand. (II) Binding energy differences. In order to implement the shape complementarity property, docked poses of the isolated ligands were compared to those in the human protein. Essentially, ligands binding different sites in Hsc70, relative to PfHsp70-x were retained for further examination.

**Figure 2:**
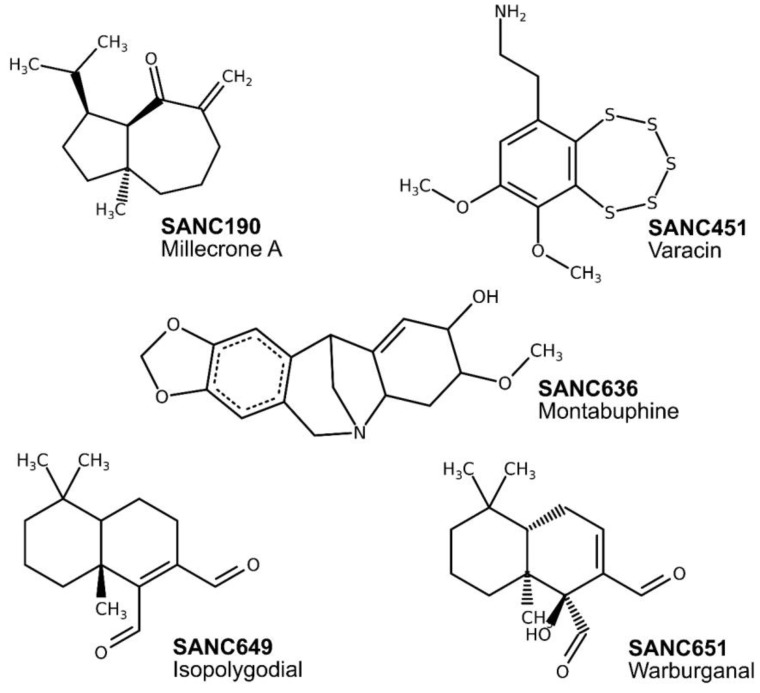
Chemical structures of identified natural compounds docked to the β-SBD back pocket in both PfHsp70-x and PfHsp70-1.

Using AutoDock4, the open conformation of PfHsp70-x, PfHsp70-1 and Hsc70 were re-screened independently against these ligands. Re-screening experiments using AutoDock4 were carried out primarily to ensure that the shape-complementarity characteristic was reproducible. Favorable ligands were selected for binding energy analysis. Following calculations of binding energy differences between parasitic and human complexes, ligands were ordered beginning with the largest energy difference. Five compounds from the top 20 positions, possessing the lowest binding energies (Table 1, Figure 2) proceeded to all atom and coarse-grained molecular dynamics simulation studies. SANC190 and SANC651 displayed considerable allosteric regulation characteristics. SANC190 (Millecrone A) and SANC651 (Warburganal) are sesquiterpenes found in extracts of the nudibranch *Leminda millecra* [63] and the stem bark of *Warburgia salutaris* [64] respectively. SANC190’s therapeutic value is still unexplored. SANC651 possesses potent antifungal and antibacterial activities [64,65]. 

### 2.4. Molecular Dynamics

**Figure 3:**
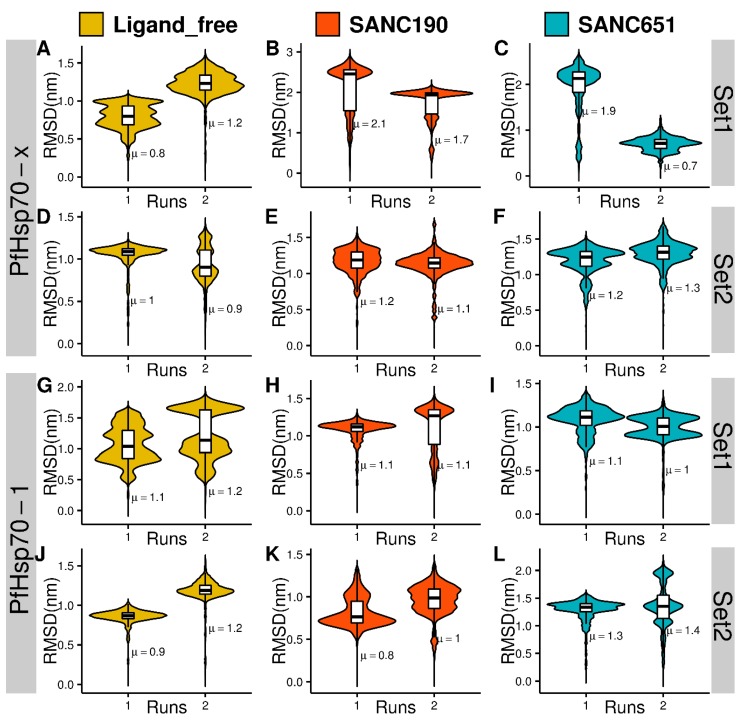
(**A**–**L**) Violin plots showing the kernel probability density distribution of protein backbone RMSDs per MD run. Density traces were plotted symmetrically to the left and right of boxplots. The width is proportional to frequency of occurrence. The overlaid boxplots highlight data range and the distribution spread. The vertical inside line represents the median value. The bars range from 25th (bottom) to 75th (top) percentile. µ denotes the calculated RMSD mean value.

**Figure 4:**
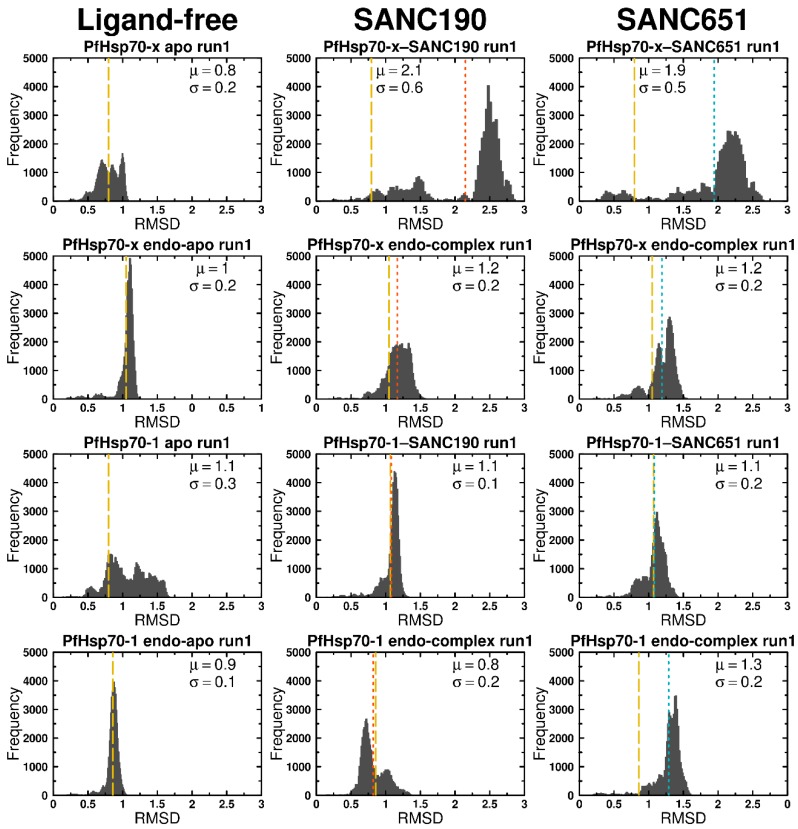
Histograms of protein backbone RMSDs against the frequency of occurrence. The distribution in conformation populations between ligand-free and ligand-bound trajectories over 100 ns were compared. Dotted lines represent positions of calculated means. Color key: Yellow: ligand-free; red: SANC190-bound; blue: SANC651-bound. Histograms of duplicate trajectories can be found in supplementary data (Figure S4).

**Figure 5:**
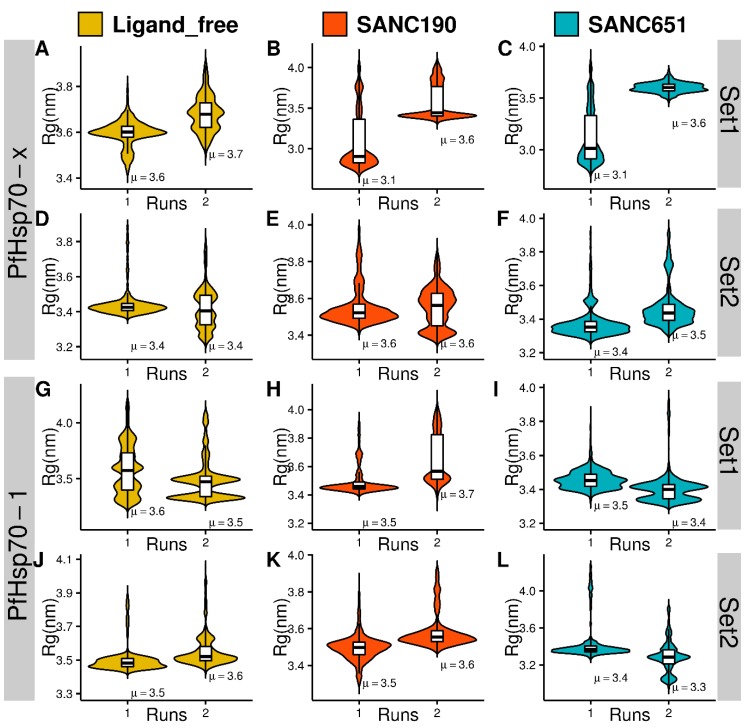
Kernel density estimation plots of the radius gyration (Rg). Comparisons of structural compactness between ligand-free and ligand-bound PfHsp70-x and PfHsp70-1. (**A**–**L**) The RMSD violin plotting scheme was employed in plotting the Rg figure as well.

One hundred nanosecond MD simulations of protein-ligand complexes gave insights into the binding effects of ligands on PfHsp70-x and PfHsp70-1. Here we present and discuss results of two ligands, SANC190 and SANC651, which showed considerable modulation effects from all-atom MD simulations. To check system stability and assess protein configurational distance from the starting conformation, RMSD calculations were carried out based on backbone atom positions. PfHsp70-1 and PfHsp70-x systems were comparatively stable at 300 K in general, converging after 50 ns around values ranging between 1.5–3.0 nm (Figure 3, Figure 4 and Figure 5, Figures S3 and S4). PfHsp70-x–SANC190 (run1 and run2) and PfHsp70-x–SANC651 (run2) recorded the largest structural deviations with RMSD values stabilizing about ~2.5 nm, ~2 nm, and ~2 nm, signifying initial conformation re-arrangements. Additionally, the trajectories yielded normal RMSD distribution patterns relative to multimodal RMSD distribution displayed by ligand-free PfHsp70-x (PfHsp70-x apo run1, run2) (Figure 3, Figure 4 and Figure 5), indicating relatively rigid systems were yielded around the equilibrium. We observed that the SBD of apo proteins (PfHsp70-x apo and PfHsp70-1 apo) moves freely during simulation. This was the case in our previous study [43]. PfHsp70-x apo, PfHsp70-1 apo, both SANC190 and SANC651 bound PfHsp70-x endo-complexes, PfHsp70-1–SANC651 complex, and SANC190 bound PfHsp70-1 endo-complex systems (Figure 3A,E,F,G,I,K) generally displayed multimodal RMSD distribution patterns suggesting that the systems yielded multiple equilibrium states during simulation. Remarkably, both PfHsp70-x endo-apo (run1) and PfHsp70-1 endo-apo (run1 and run2) yielded normal RMSD distribution patterns indicating that the inclusion of endogenous ligands tends to relax the systems. Despite inconsistencies in average RMSD values between runs, the extent to which duplicate simulations diverged was small, therefore meaningful conclusions could be inferred from trajectories. Altogether, ligand-free systems, SANC190 and SANC651 bound PfHsp70-x endo-complex, PfHsp70-1–SANC651 complex, SANC190 and SANC651 bound PfHsp70-1 endo-complex displayed minimal deviation from the initial structure compared to PfHsp70-x-SANC190, PfHsp70-x-SANC651 and PfHsp70-1-SANC190 complexes during simulation.

The RMSF calculations revealed local mobility properties of residues on average. First, nearly similar patterns of residue fluctuations were evident across systems: Residues positions 225–255 and 275–300 (NBD), 375–400 (conserved linker region), 500–525, 550–575, 585–606 (SBD) generally exhibited large fluctuations (Figure S5). Secondly, Set2 systems displayed appreciable smaller values of residue fluctuations compared to Set1 systems globally, indicating that the presence of endogenous ligands reduces structural mobility to some degree. Relative to ligand-free trajectories, PfHsp70-x–SANC190 complex (run1 and run2) and PfHsp70-1–SANC651 complex (run1 and run2) exhibited increased and decreased fluctuations respectively throughout.

We also compared the dynamic profiles for studied systems obtained from all-atom and coarse-grained simulations. CABS-CG simulations of the studied systems revealed very similar general trends (Figure S10). By comparing profiles of the computed B-factors for the apo forms of PfHsp70-1 and PfHsp70-x proteins, we found that PfHsp70-x apo form is somewhat more stable, while both trajectories showed significant variations in the NBD regions (residues 225-255, 275-300), the linker region and SBD fragments (residues 500-520, 550-575) (Figure S10, panel A). Consistent with the all-atom MD simulations, apo forms of the PfHsp70-1 and PfHsp70-x proteins in the presence of endogenous ligands (Set 2) exhibited the markedly reduced fluctuations (Figure S6, panel B). The observed stabilization effect of endogenous ligands is in line with the all-atom MD results, but CG simulations showed a more significant ligand-induced effect. This may reflect the energetic preferences caused by a more limited set of allowed local moves and geometric restrictions as compared to all-atom simulations that are more sensitive to different degrees of protein plasticity. In the absence of endogenous ligands, the apo form of PfHsp70-x was more stable. This trend was also observed in simulations with the bound endogenous ligands as both apo forms showed considerable stabilization (Figure S6, panels A,B). These findings are consistent with all-atom simulations, showing that CG approach is fairly robust in revealing the general dynamic patterns and identifying rigid and flexible regions in the proteins. The addition of ligands (SANC195 and SANC651) to a more flexible PfHsp70-1 (Set1) results in the reduction of conformational variations that is broadly distributed in the protein structure (Figure S6, panel C). In other words, these ligands seem to exert an appreciable allosteric effect over the long distance and confer a more stable protein structure. CABS-CG simulations of PfHsp70-x complexes with these ligands showed a very moderate increase in structural mobility, suggesting that the effect of allosteric ligands may marginally counteract the stabilization induced by endogenous ligands in the PfHsp70-x endo-complexes. Overall, we observed that binding of SANC190 and SANC651 ligands can affect conformational dynamics of PfHsp70-x and PfHsp70-1 proteins, and this allosteric effect can be delicately balanced by the endogenous ligands. The radius of gyration (Rg) was calculated to assess compactness of models as a function of time. Rg values ranged between 3.1–3.7 nm in general across models (Figure 5). Ligand-free trajectories recorded Rg values 3.4–3.7 nm on average. PfHsp70-1 endo-apo (run1 and run2), PfHsp70-x endo-apo (run1), and PfHsp70-x apo (run1), displayed normal distributions of Rg value while other ligand free systems displayed multimodal distribution patterns. Compared to ligand-free systems, PfHsp70-x–SANC190 (run1), PfHsp70-x–SANC651 (run1) registered the lowest average Rg values (3.1 nm each) suggesting increased structural compactness due to ligand binding. Overall, while ligand-free systems exhibited nearly symmetric Rg spread patterns, indicating relatively maintained compaction-expansion layouts, PfHsp70-x–SANC190 complex (run1, run2), PfHsp70-x–SANC651 (run1), SANC190-bound PfHsp70-x endo-complex (run1, run2), PfHsp70-1–SANC190 complex (run1, run2) displayed skewed Rg distribution patterns suggesting ligand-specific Rg deviation from the native fold.

### 2.5. Essential Dynamics

**Figure 6:**
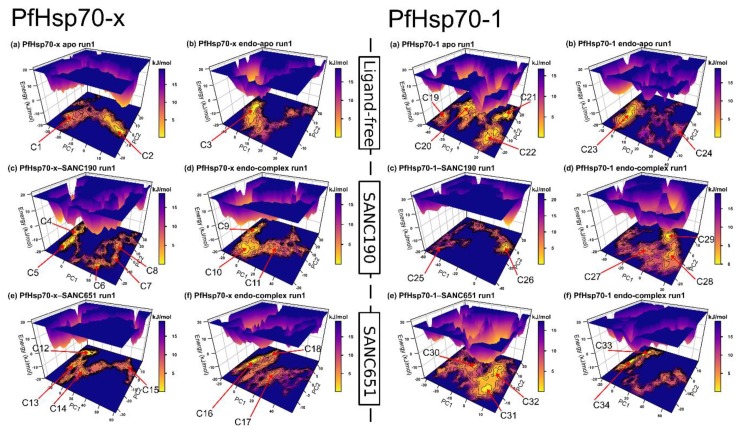
Gibbs free energy landscapes illustrating conformation equilibrium observables as a function of PC1 and PC2. Colors range from yellow (Gibbs free energy minima) to purple (Gibbs free energy maxima). Each contour ring represents a change in the Gibbs free energy by 1 kJ/mol. Conformations visited (conformers) during simulations were labeled from C1–C30.

Trajectories were visually inspected using VMD [66]. PfHsp70-x–SANC190 and PfHsp70-x–SANC651 trajectories displayed large structural repositioning motions: The binding of ligands bring both domains (SBD and NBD) close to each other. This was clearer in PfHsp70-x–SANC190 run1 and PfHsp70-x–SANC651 run1 (Video S1, Video S2, Video S3). To explain the above interdomain advances, principal component analysis (PCA) calculations were performed on trajectories. PCA allows extraction of large-scale important motions that describe conformation ensembles as well as redistribution patterns occurring during simulation in subspaces of reduced dimensionality [67]. From a total of 1818 eigenvectors, PC1 and PC2 captured at least 55% of the total variance (Table S3), indicating that the top two components were sufficient to describe predominant protein motions. To get a detailed illustration of PfHsp70-x and PfHsp70-1 conformational sampling around ligand-free and ligand-bound states, free energy landscapes (FEL) were calculated, and projected as a function PC1 and PC2. It is thought that population shift patterns of conformational states could be linked to functional changes associated with allosteric modulation [68]. The FEL provides a 3D depiction of conformation clusters around their free-energy sub-states allowing evaluation of ensemble convergence. Comparing ligand-bound and ligand-free trajectories between sets (Set1 and Set2), Set1 trajectories generally occupied well defined energy basins compared to Set2 at 300 K (Figure 6, Figure S7), suggesting that the incorporation of endogenous ligands in Set2 confers a relaxation effect on structural dynamics.

**Figure 7:**
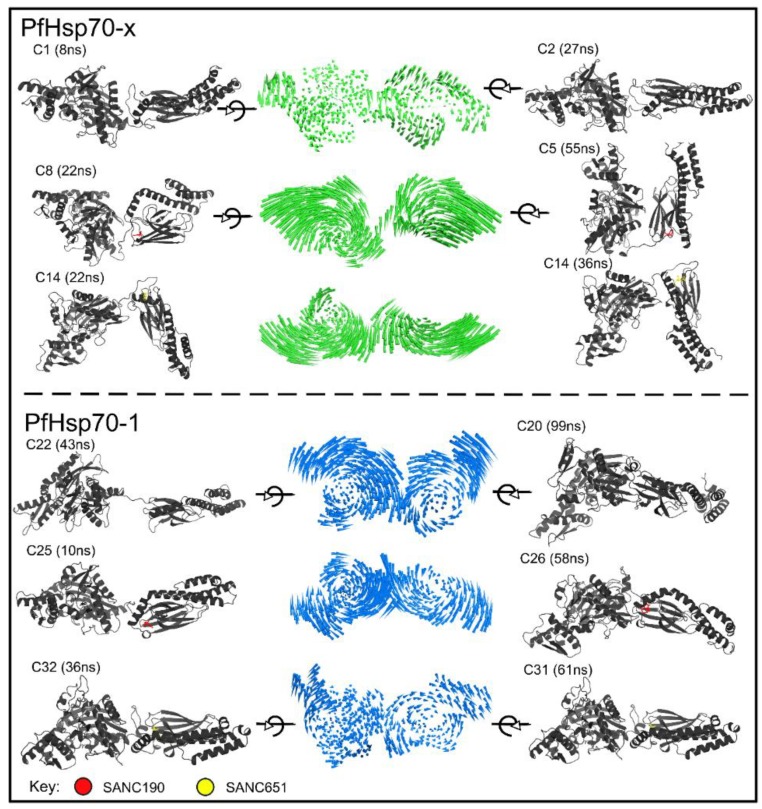
Representative ligand-free and ligand-bound sub states (colored grey) of PfHsp70-x and PfHsp70-1. Structures were obtained from conformation clusters occupying low energy basins as depicted in Figure 6. The middle panel displays porcupine plots illustrating the direction and magnitude (indicated by the length of the porcupine) of dominant protein motions observed during simulation. Color code: Green: PfHsp70-x, Blue: PfHsp70-1. Circular arrows indicate ~90° rotation of structures.

Besides, PfHsp70-1 apo (run1: Four conformers, run2: Four conformers), ligand-free systems, including PfHsp70-x apo (run1: Two conformers, run2: Two conformers), PfHsp70-x endo-apo (run1: One conformer, run2: One conformer) and PfHsp70-1 endo-apo (run1: Two conformers, run2: Two conformers) populate fewer energy basins, however, span a broader conformational space suggesting relatively stable ensembles. With respect to Figure 6, the binding of SANC190 and SANC651 to PfHsp70-x strongly influences the mobility and conformation re-distribution patterns of Set1 systems. Both PfHsp70-x–SANC190 and PfHsp70-x–SANC190 produce a series of folding funnels characterized by low lying free-energy barriers, suggesting ligand-specific regulation of the folding process. Indeed, both models project enhanced structural activities in both domains (NBD and SBD) (Figure 7): The binding of ligands triggers interdomain advances about the linker (linker acts as a hinge). Although in opposite directions, (Figure 7, Video S2, Video S3), such ligand-induced domain advances are also reported in the nucleotide-dependent Hsp70 functional cycle [15]: The binding of ATP is thought to induce global structural re-arrangements about the linker promoting the formation of an allosterically active NBD-SBD interface. Higher trace values of associated trajectories (PfHsp70-x–SANC190: 1728.48, and PfHsp70-x–SANC651: 1631.52) (Table S3) indicate increased structural flexibility of PfHsp70-x, which could be explained by conformation rearrangement events taking place during simulation. On the other hand, SANC190 and SANC651 induce structural rigidity to a more flexible PfHsp70-1 (Set1) as shown in Figure 7. Additionally, both systems visit fewer metastable states (PfHsp70-1–SANC190: Two conformers, PfHsp70-1–SANC651: Three conformers) on the free energy landscape. Lower flexibility characteristics, as depicted by corresponding low trace values (PfHsp70-1–SANC190: 494.78, PfHsp70-1–SANC651: 225.78, to PfHsp70-1 apo: 821.34) (Table S3), could be explained by the fact that both complexes displayed paltry structural re-arrangements during simulation.

Taken together the results and analysis of all-atom and CG simulations, it was evident that SANC190 and SANC651 strongly modulate conformational dynamics of PfHsp70-x and PfHsp70-1 and could consequently regulate succeeding ADP/substrate release events. While both ligands induced substantial structural re-adjustments on PfHp70-x (considering Set1 results), structural rigidity was conferred to a more dynamic PfHsp70-1. These findings were also supported by CG simulations, offering further support to our conclusions.

### 2.6. Thermodynamic Assessment

To better understand the solidity of protein-ligand interactions, the MMPBSA [69] approach was used to compute binding free energies of protein-ligand complexes. MMPBSA method has proven to be valuable and computationally cost-effective in estimating relative binding free energies of ligands to macromolecules. Based on RMSD equilibrium profiles, protein-ligand binding free energies were computed as an average of trajectory snapshots sampled in the last 15 ns at 10 ps intervals. Overall, both SANC190 and SANC651 bound PfHsp70-x and PfHsp70-1 complexes recorded low total binding free energy values ranging between −104.929 kJ/mol and −56.488 kJ/mol (Table S4), indicating robust intermolecular associations. SANC190 yielded lower energy values to both PfHsp70-x and PfHsp70-1 compared to SANC651 in general. Across models, the van der Waal (ΔE*_vdW_*) and polar solvation (ΔG*_polar_*) energy terms contributed the lowest and highest values respectively to the total binding free energy.

### 2.7. Dynamic Residue Network Analysis

The effects of ligand binding on intraprotein communication were examined by performing DRN analysis on ligand-free and ligand-bound systems. RIN calculations have been used previously to effectively identify key functional residues that adjudicate protein folding [70,71,72,73]. RIN graphs were constructed using MD-TASK toolkit [74]. The communication value of each residue was evaluated quantitatively based on two important metrics: (1) The average shortest path (*L*), and (2) betweenness centrality (*BC*). *L* and *BC* have proved successful at identifying critical communication hubs that could regulate allosteric transitions in proteins [45,73,75]. *L* highlights the spatial placement of a residue, such that it is conveniently obtainable for communication. Low *L* values refer to readily accessible nodes/residues, and the vice-versa is true. *BC* highlights the magnitude of importance of a residue in a communication network by measuring how often the residue is visited along shortest paths of all other residues. High *BC* values correspond to highly connected nodes/residues, thus critical communication hubs. DRN describes the moving average of *L* (average *L*) and *BC* (average *BC*) calculated over a trajectory.

We noted that the global distribution of average *L* values across models (both ligand-free and ligand-bound PfHsp70-x and PfHsp70-1) was generally similar (Figure S8). Likewise, threshold values (Table S5) applied to identify low average *L* regions across systems suggested that the overall topological layout, with respect to average *L* dips, was fairly maintained. In regard to global adjustments due to ligand binding, ligand-bound PfHsp70-x Set1 systems (PfHsp70-x–SANC190, PfHsp70-x–SANC651) recorded lower threshold values compared to the ligand-free system (PfHsp70-x apo) on average, indicating a general decrease in average shortest path lengths. On the contrary, ligand-bound PfHsp70-x Set2 models (SANC190-bound PfHsp70-x endo-complex, SANC651-bound PfHsp70-x endo-complex) registered higher threshold values, relative to the ligand-free system (PfHsp70-x endo-apo) on average, indicating that the presence of endogenous modulators influences, to some extent, global rearrangements in average *L*. This observation was reproduced in SANC651-bound PfHsp70-1 models which yielded lower (Set1) and higher (Set2) values, relative to ligand-free PfHsp70-1 systems (apo and endo-apo respectively) (Table S5). On the other hand, SANC190-bound PfHsp70-1 systems (Set1 and Set2) yielded lower threshold values relative to ligand-free PfHsp70-1 systems (Set1, Set2), indicating that the presence of endogenous modulators negligibly influence average *L* adjustments in these systems. Based on ligand-free profiles of both PfHsp70-x and PfHsp70-1, residues 1–17, 22–25, 34–36, 66–67, 117–129, 136–144, 155–159, 162–179, 365–380 possessed low values in general, demonstrating that in the native state, these regions are highly reachable for communication.

In relation to average *BC* profiles, PfHsp70-x and PfHsp70-1 ligand-free models displayed subtle differences in the distribution of peaks in general (Figure S9A,B), suggesting that both proteins innately possess nearly similar communication arrangements. Based on these profiles, residues 36, 120, 121, 164–167, 335, 367, 371, 381, 385 (NBD), 396 (linker), 407–412, 441, 445, 452, 478, 501–507, 517, 534–538 (SBD) of PfHsp70-x, and residues 3, 5, 10, 36, 120–129, 135, 136, 164, 165, 170, 274 (NBD), 407, 411, 419, 475–477, 481–485, 510, 513, 535 (SBD), of PfHsp70-1 generally yielded large betweenness indices, indicating the residues predominantly participate in cross-domain communication. Interestingly, the network analysis and *BC* profiles derived from CG simulations revealed not only similar overall trends but also pointed to the same functional regions and specific residues corresponding to BC profile peaks (Figure S10). The comparative *BC* profiles for PfHsp70-x and PfHsp70-1 proteins in the absence and presence of endogenous ligands were quite similar with the conserved *BC* peaks largely unchanged. The quantitative agreement between *BC* profiles obtained from all-atom and CG simulations may be explained by the topological nature of the network-based indexes. Indeed, the salient aspects of the network organization in protein structures and high centrality sites are mainly determined by the topological architecture of the fold which can be accurately captured in CG simulations. 

Besides SANC651-bound PfHsp70-x endo-complex (Set2), SANC651-bound PfHsp70-1 endo-complex (Set2), SANC190-bound PfHsp70-1 endo-complex (Set2), all ligand-bound models recorded lower threshold values (Table S5), compared to corresponding ligand free systems indicating a general decrease in average *BC* values because of ligand binding. To assess local effects of ligand binding on residue interaction network layout, ligand-free and ligand-bound trajectories were compared by calculating per residue differences in average *L* and average *BC* indices. 

First, we illustrated in the past that there exists strong positive correlation between *L* and RMSF [42,43], and moderate and weak inverse correlation between *BC* and *L* or RMSF, respectively [43]. These findings were specific to highly dynamic proteins. Here, we support these observations by reporting nearly similar findings (Table S6). Following calculations using pairwise Pearson’s correlation coefficient, average *L* and RMSF were highly correlated (r range = 0.41–0.81), *BC* and *L*^−1^ displayed intermediate linear relationship (r range = 0.28–0.55), while *BC* and RMSF yielded low values indicating poor correlation (r range = 0.03–0.23).

**Figure 8:**
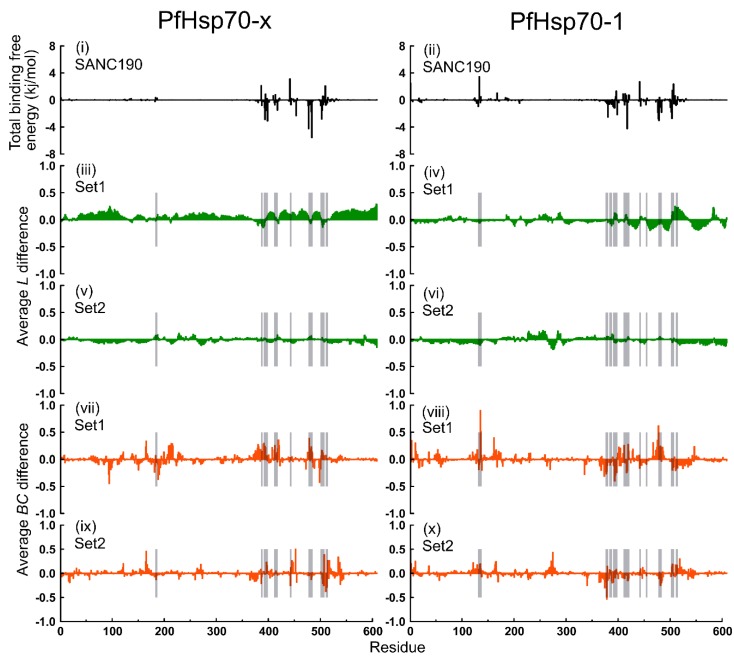
Per residue average *L* and average *BC* difference calculated between ligand-free and SANC190-bound systems (run1 and run2 on average). Top panel: Residues governing protein-ligand affinity; middle panel: Per residue average *L* difference; bottom panel: Per residue average *BC* difference.

**Figure 9:**
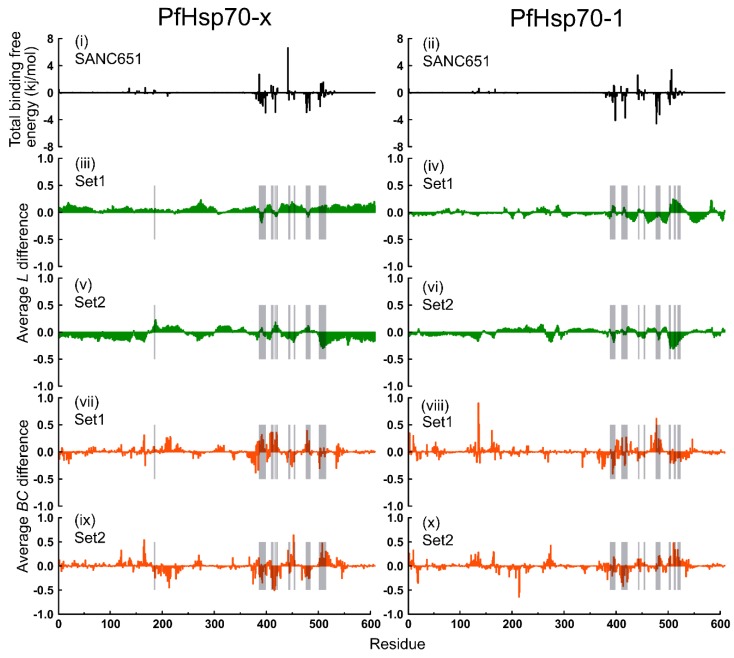
Computed average *L* and average *BC* per residue difference between ligand-free and SANC651-bound models (run1 and run2 on average). Top panel: Residues governing protein-ligand affinity; middle panel: Per residue average *L* difference; bottom panel: Per residue average *BC* difference.

Secondly, previous reports indicate that global changes in average shortest path (*L*) positively correlate to adjustments in the radius of gyration (Rg) [76]. Recently, we complemented these findings by showing that ligand-invoked spatial expansion/compaction of protein structures in relation to the center of mass could influence shifts in average *L* values of the entire protein length, but ligand–binding regions [43]. Opposition by ligand-binding sections to widespread average *L* adaptation inversely modifies reachability thereof [43]. These observations were replicated in our current experiments. While ligand-bound PfHsp70-x and PfHsp70-1 displayed global collective shifts/changes in average *L* profiles, especially in the target domain (SBD), ligand-binding regions expressed minimal to no changes as expected (Figure 8 and Figure 9). From calculated means of average *L* difference between ligand-bound and ligand-free systems, PfHsp70-x–SANC190 (0.072) and PfHsp70-x–SANC651 (0.656) registered positive values, while all other systems including PfHsp70-1–SANC190(−0.018), PfHsp70-1–SANC651(−0.009), SANC190 bound PfHsp70-x (−0.016) and PfHsp70-1 (−0.0165) endo-complexes, SANC651-bound PfHsp70-x (−0.047) and PfHsp70-1 (−0.013) endo-complexes registered negative values; meaning ligand binding regions of PfHsp70-x–SANC190 and PfHsp70-x–SANC651 recorded decreased reachability, while the vice-versa holds for other systems.

Previously, we also illustrated that ligand binding can generate wide-scale re-organization of betweenness indices leading to either increased or decreased numbers of recognized betweenness peaks [43]. Increased numbers of betweenness peaks can correspond to decreased threshold values and may indicate loss of significant betweenness peaks. On the other hand, decreased numbers of residues with high betweenness indices can correspond to increased threshold values and may indicate emergence of non-native significant peaks. Gain or loss of significant betweenness peaks consequently modifies the native information flow network. We proposed that the ensuing modifications could eventually determine ligand-specific modes of action. Besides residues participating in protein-ligand interactions, residues exhibiting large changes in betweenness centrality, particularly residues with known function-related properties, could aid in explaining consequent mechanistic routes of ligand-induced allosteric activation [43]. 

Average *BC* changes, unlike average *L* changes, are more localized. Notably, there was high overlap between residues that largely govern protein-ligand affinity, including neighboring residues, and residues that registered large changes in average *BC* in general (Figure 8 and Figure 9). Besides ligand binding sections, distant regions within the NBD displayed large changes in average *BC* values suggesting far-reaching consequences from long-range contacts. In general, residue positions 94, 121, 164, 165, 188, 213, 390, 391, 419, 445, 452, 478, 498, 507, 534 (PfHsp70-x) and 3, 10, 129, 135, 136, 161, 165, 274, 373, 374, 377, 378, 393, 394, 477 (PfHsp70-1) of SANC190-bound systems and residues 121, 164, 165, 374, 378, 379, 384, 415, 417, 419, 452, 507, 517, 534 (PfHsp70-x), and 10, 129, 135, 136, 161, 165, 274, 419, 477, 481, 213, 214, 373, 379, 413, 511, 514, 534 (PfHsp70-1) of SANC651-bound systems exhibited large changes in betweenness indices. This evidently implies that ligand binding may not only modulate the kinetics of pre-bound substrate, but also the dynamics of ADP. 

In regard to global motions results discussed earlier (Video S1, Video S2) (refer to essential dynamics section), it was clear that SANC190 and SANC651 binding to PfHsp70-x (Set1) triggers structurally large conformational rearrangements. In both cases, the ligands generate enhanced conformational flexibility (Table S3) effectively leading to interdomain advances about the linker. Natively, ATP binding activities, including consequent perturbations at the NBD are thought to induce interdomain advances, coupling and eventual NBD-SBD docking [14,18]. It is likely that the binding of SANC190 and SANC651 could induce similar agonistic properties accelerating conformation transition kinetics from the closed state to the more compact open state. We found that a considerable number of residues displaying large changes in betweenness index correspond to potential allosteric communication hubs thought to promote opening conformational transition in *E. coli* Hsp70 when force perturbations were applied [11] (Table S7). It should be pointed out that the proximity of the binding site to the conserved interdomain linker may have an important role to play in dictating conformational plasticity. Among residues that displayed substantial changes in average *BC*, VAL393, CYS394 LEU483, ASN502, of PfHsp70-x–SANC190 complex and LYS386, VAL393, CYS394, LEU453, ASP478, ILE482, ASN502, of PfHsp70-x–SANC651 complex correspond to residues either making molecular interactions with the ligand (Figure S11), or governing protein-ligand binding affinities (Tables S7 and S8). Notably, average *BC* changes affecting residues LEU390, LEU391, VAL393, CYS394, (in both SANC190 and SANC651 bound PfHsp70-x) located in the conserved interdomain linker implicated in cross-domain allosteric regulation [12], could play an integral role in facilitating the observed conformational switches. Additionally, average *BC* alterations registered in hinge bending residues SER383, VAL385 [73,77], PRO418, THR419, LYS420 [18] (of SANC190-bound) and THR416, PRO418, THR419, LYS420, TYR440, LEU453 [18] (of SANC651-bound) (Table S7) could complement the observed intradomain re-positioning activities. Following assessment of global and local protein dynamics, we can conclude that SANC190 and SANC651 strongly portray allosteric activation (agonism) attributes on the closed conformation PfHsp70-x protein, wherein ligand binding stimulates domain advances comparable to proceeding innate ATP binding events.

## 3. Materials and Methods

### 3.1. Data Retrieval and Phylogenetic Tree Analysis

PfHsp70-x sequence (accession number: PF3D7_0831700) was obtained from PlasmoDB database (http://plasmodb.org/plasmo/). Protein Basic Local Alignment Search Tool (BLASTP) was used to search and retrieve PfHsp70-1 (accession number: XP 001349336.1), Hsc70 (accession number: AAK17898.1), 7 non-falciparum (plasmodial), 19 Protozoan (non-plasmodial), and 19 Eukaryotic (non-protozoan) homologous sequences from National Center for Biotechnology Information database (NCBI) (http://www.ncbi.nlm.nih.gov/ ). Reverse-BLAST was carried out against each of the sequences retrieved to establish true orthologs (Table S1). Multiple sequence alignment was performed using PROMALS3D [78] with default parameters. The alignment input incorporated sequences extracted from Protein Data Bank (PDB) structures 2KHO and 4B9Q of *E. coli* origin. A phylogenetic tree was constructed using MEGA6 [79]. 2KHO and 4B9Q chain A sequences were used as outgroups. Calculation of best-fit substitution model and phylogeny reconstruction was performed using maximum likelihood statistical method. Since Hsp70 sequences are well conserved across species, we rationalized complete gap deletion. A total of 1000 bootstrap replications were performed. The appropriate phylogeny was identified from the top three substitution models (Le and Gascuel: Gamma distributed (LG+G) and Gamma distributed with invariant sites (LG+G+I) [80], and reverse transcriptase (rtREV+G+F) [81]) which yielded the lowest BIC (Bayesian Information Criterion) scores.

### 3.2. Homology Modeling

Due to lack of experimental three-dimensional (3D) structures of target proteins PfHsp70-x, PfHsp70-1, and Hsc70, in the Protein Data Bank (PDB), protein homology modeling technique was employed. Identification of suitable templates was done using PRIMO web server [47]. Default template recognition and alignment options were used. Related *E. coli* structures PDB ID: 2KHO (closed conformation) and 4B9Q_chain A (open conformation) were identified and respective sequences retrieved. Template-target alignment was performed using PROMALS3D tool [78]. To increase the alignment accuracy, multiple sequence alignment incorporating the 45 retrieved close homologues was performed as described earlier. PfHsp70-x, PfHsp70-1, and Hsc70 sequences were each aligned to each template structure sequence (2KHO and 4B9Q) generating six MODELLER [51] alignment .PIR files: Three for open conformation models and three for closed conformation models. For each alignment profile, 100 models were built using MODELLER version 9.12 [51] with slow refinement. In total, 600 models were generated. Structural qualities of obtained models were assessed both as a whole and at local level. Initially, individual models were ranked based on normalized z-DOPE (Discrete Optimized Protein Energy) score [52]. The top three models advanced to local evaluation using PROCHECK Ramachandran plot [53], and VERIFY3D [54] tools. Following a consensus of the three assessment tools, six top quality structures per protein conformation (PfHsp70-x, PfHsp70-1, and Hsc70 in both closed and open conformations) were identified and used for docking simulation studies.

### 3.3. Molecular Docking

High throughput virtual screening (HTVS) allows rapid identification of a subset of active compounds from a large collection of potential modulators. HTVS was performed on open and closed conformation models of PfHsp70-x, PfHsp70-1 and Hsc70 in order to identify *P. falciparum* selective hits. Each protein conformation was screened against 623 compounds retrieved from the South African Natural Compound Database (SANCDB) [82]. Receptors and ligands were prepared using Autodock4 tools utilities [56]. A rigid-protein flexible-ligand docking protocol was applied. Default torsional degrees of freedom were set in ligands and Gasteiger atomic charges assigned. Docking simulations were carried out using both AutoDock Vina [55] and AutoDock4 [56] (ADT). To validate the docking protocol, reproducibility of co-crystallized poses was initially assessed. For the closed conformation, validation was performed on PDB ID: 3ATV structure by re-docking co-crystallized ADP. For the open conformation, co-crystallized ATP was re-docked to 4B9Q structure. Docking parameters specified for each docking program below were applied. 

At first, docking experiments were performed using AutoDock Vina. (1) Open conformation: Blind docking was performed on each protein. A grid box search space of dimension size: *x* = 48.0 Å, *y* = 62.0 Å, *z* = 80.0 Å was defined. Ligands were centered at *x* = 106.0, *y* = 76.0, *z* = 99.0. A search exhaustiveness value of 512 was applied. (2) Closed conformation: Entire surfaces of each protein were screened as well. For a more focused and computationally efficient docking simulation, two grid boxes concentrating on each domain were used. Grid boxes of dimension sizes: *x* = 48 Å, *y* = 62 Å, *z* = 80 Å enclosing the NBD and *x* = 36 Å, *y* = 48 Å, *z* = 75 Å enclosing the SBD, with some overlap on the interdomain linker region were defined. Ligands were centered at *x* = 0.0, *y* = 0.0, *z* = 0.1 (NBD), and *x* = 0.0, *y* = −9.7, *z* = −63.2 (SBD) respectively. An exhaustiveness value of 512 was set throughout. For each docking trial, ten ligand poses were generated and ranked using Vina scoring function.

Next, heatmaps were used to identify the more promising parasitic target protein and conformation, as well as the more ligand-receptive target domain of the prioritized protein conformation. The SBD of the closed conformation of PfHsp70-x was identified. Also, two potential allosteric ligand-binding sites were identified in PfHsp70-x (the β-SBD back pocket, and a β-SBD–A-LID interface) (Figure S2).

Thirdly, AutoDock4 was used to re-screen the entire surface of the open conformation of PfHsp70-x against ligands isolated from the above binding sites. Also, PfHsp70-1 and Hsc70 open conformations were re-screened for comparison purposes. The following docking parameters were applied: (1) Two grid boxes were defined: Size *x* = 84.0 Å, *y* = 108.0 Å, *z* = 126.0 Å enclosing the NBD and size *x* = 84.0 Å, *y* = 74.0 Å, *z* = 112.0 Å enclosing the SBD, with some overlap on the interdomain linker. (2) Ligands were centered at *x* = 0.0, *y* = 0.0, *z* = 0.0 (NBD) and *x* = 0.0, *y* = −9.7, *z* = −61.0 (SBD) respectively. The Lamarckian genetic algorithm (LGA) was implemented. A population size of 150 was set and a maximum of 10,000,000 energy evaluations carried out per LGA run. A total of 100 docking trials were performed, and free energy of interaction computed using AutoDock4 scoring function. Docked poses were ranked based on the lowest binding energies in the largest cluster. 

Finally, compounds were ordered based on shape complementarity and binding energy characteristics; (I) favorable hits were bound to distinct pockets in parasitic proteins relative to the human protein; (II) ligand-binding sites were strictly reproduced by AutoDock4; (III) best “hits” interacted with lower binding energies to parasitic proteins compared to human proteins. The top five compounds (Table 1) that yielded the most negative binding energies were retained for all atom and coarse-grained MD simulation experiments.

### 3.4. All Atom Molecular Dynamics Simulations

All atom molecular dynamics calculations were performed on protein-ligand complexes using GROMACS 2016.4 [83] at the Centre for High Performance Computing (CHPC), Cape Town. An experimental design similar to our previous study [43] was implemented. Throughout the text, we will refer to exogenous ligands (SANC190 and SANC651) as “ligands”. The dynamics of each protein (PfHsp70-x and PfHsp70-1) was studied against each ligand. We will refer to ligand-free models as “apo” and ligand-bound as “complex”. An “endo” prefix will be used to refer to systems consisting endogenous modulators (ADP and peptide substrate). Two sets of experiments were studied: Set1 involving protein models less endogenous modulators (PfHsp70-x apo, PfHsp70-x-ligand complex, PfHsp70-1 apo, and PfHsp70-1-ligand complex) and Set2 including protein models consisting of endogenous modulators (PfHsp70-x endo-apo, PfHsp70-x endo-complex, PfHsp70-1 endo-apo, PfHsp70-1-endo-complex). All calculations utilized the AMBER03 force-field [84]. Models were placed in a triclinic box with 2 Å buffering distance around the protein. The solute was hydrated with explicit simple point charge (SPC126) water molecules and neutralized with 0.15 M Na^+^ and Cl^−^ counter-ions. Fifty thousand steps of relaxation using the steepest descent method were applied without position restraints on solute atoms, until the systems converged to a force threshold value of ≤1000 kJ/mol/nm. Systems were equilibrated to a temperature of 300 K at constant volume (NVT) using the modified Berendsen thermostat. Afterwards, systems were equilibrated at 1 bar pressure and constant temperature and volume (NPT) using the Parrinello-Rahman barostat. In both ensembles, two coupling groups (protein and non-protein) and coupling time constants of 0.1 ps and 2.0 ps respectively were set, all bonds lengths were fixed using the Lincs constraint algorithm, and equilibrations runs performed for 200 ps at 2 fs time steps. Treatment of long-range electrostatics was conducted using the Particle Mesh Ewald (PME) method. Van der Waals interactions were computed using Lennard Jones potential. Cut-off distance values of 1.4 nm were applied for non-bonded interactions. Finally, restraints were removed and periodic boundaries in all directions set. Multiple production calculations of 100 ns with 0.002 ps timestep starting with different initial velocities were carried. System co-ordinates were written every 2 ps.

### 3.5. Coarse-Grained Monte Carlo Dynamics Simulations

We also performed coarse-grained (CG) simulations of studied systems using CABS CG model for simulations of protein structures and dynamics [85,86]. In this CG model, protein structures the amino acid residues are represented by four united atoms, namely main-chain α-carbons, β-carbons, the center of mass of side chains and another pseudoatom placed in the center of the Cα-Cα pseudobond. The sampling protocol of the CABS-CG model is primarily based on Monte Carlo (MC) dynamics approach and involves a random sequence of small local moves of individual amino acids in the protein structure as well as moves of small fragments composed of 2–3 amino acids [85,86,87]. It has been previously shown that CABS MC dynamics trajectories can accurately recapitulate the all-atom MD simulations for long-time processes [86,87,88]. The simulations were carried out with the new CABS-flex standalone Python package for fast simulations of protein dynamics implemented as a Python 2.7 object-oriented package [89]. Similar to all-atom MD simulations, CABS-CG simulations were done for Set1 (PfHsp70-x apo, PfHsp70-x-ligand complex, PfHsp70-1 apo, and PfHsp70-1-ligand complex) and Set2 (PfHsp70-x endo-apo, PfHsp70-x endo-complex, PfHsp70-1 endo-apo, PfHsp70-1-endo-complex). Only two exogenous ligands (SANC190 and SANC651) were used for ligand-protein complex simulations.

### 3.6. Preliminary Analysis of Trajectories

GROMACS utilities were used for preliminary analysis of trajectories. To assess simulation qualities, potential energy, kinetic energy, and temperature values were examined. Periodic boundaries were eliminated, and systems centered on the protein using *gmx trjconv* module. The root-mean-square deviation (RMSD), root-mean-square fluctuation (RMSF), and radius of gyration (Rg) were computed using *gmx rms*, *gmx rmsf*, *gmx gyration* modules.

### 3.7. Essential Dynamics

Principal component analysis (PCA) was carried out in order to gain insights into the accessible conformation space of PfHsp70-x and PfHsp70-1, and the associated conformation re-distribution patterns because of ligand-binding. PCA allows reduction of dimensionality of trajectories and visualization of collective motions on a lower subspace. The covariance matrix was built based on *Z* backbone atoms positions using GROMACS tools (*gmx covar*). Briefly, for a trajectory with a set of *p* observations {Z_1_, …, Z_p_}, the covariance, denoted *C*, is a 3*Z*X*Z* matrix in which an element $C_{mn}$ is estimated by:

(1) $$C_{mn}=\left(P_{m}-{\bar{P}}_{m}\right)\left(P_{n}-{\bar{P}}_{n}\right)$$

where *m* and *n* represent values of each of the 3*Z* cartesian co-ordinates, ${\bar{P}}_{m}$ and ${\bar{P}}_{n}$ represent the average values across observations, $\left(P_{m}-{\bar{P}}_{m}\right)$ and $\left(P_{n}-{\bar{P}}_{n}\right)$ represent the positional deviation of observations from the mean. Using *gmx anaeg*, the covariance was decomposed into a transformation matrix made up of eigenvectors sorted in descending order of associated eigenvalues. Eigenvectors corresponding to the top two elements (PC1 and PC2) were obtained for analysis. Generally, most of the variance is expressed by PC1 and PC2. Ensemble re-distribution patterns during simulation were analyzed by examining free energy landscapes (FEL). The FEL as a function of PC1 and PC2 were computed using *gmx sham* module.

### 3.8. Binding Free Energy Analysis

Relative binding free energies of protein-ligand complexes were calculated using Molecular Mechanics Poisson Boltzmann Surface Area (MMPBSA) method [69] implemented in the *g_mmpbsa* module [90]. Based on RMSD equilibrium profiles, the average ΔG values were calculated on 7500 trajectory snapshots spanning the last 15 ns, sampled at 10 ps intervals. Generally, protein-ligand binding free energy can be estimated by the equation:

(2) $$\Delta G_{binding}=\Delta G_{complex}-\left(\Delta G_{protein}-\Delta G_{ligand}\right)$$

where $\Delta G_{complex}$, $\Delta G_{receptor}$ and $\Delta G_{ligand}$ represent Gibbs-free energies of endpoint states: The protein-ligand complex (bound), unbound protein, and free ligand in that manner. The Gibbs-free energy value of each component, denoted $\Delta G_{x}$, can be calculated from changes in molecular mechanics energy (Emm), entropic potential (T: Temperature, S: Entropy), and solvation free energy as follows:

(3) $$\Delta G_{x}=Emm-\left(TS+G_{solv}\right)$$

Practically, *g_mmpbsa* tool computes relative binding free energy from Emm, and $G_{solv}$ energy terms (Equation (4)). Emm: Vacuum potential explaining bonded and non-bonded interactions, $G_{solv}$: Polar (computed by solving the Poisson Boltzman equation), and non-polar (estimated from the solvent accessible surface area (*SASA*)) solvation free energy.

(4) $$\Delta G_{x}\approx Emm-\left(TS+G_{solv}\right)$$

Total binding free energies were decomposed on per residue basis to obtain quantitative insights into the critical protein residues involved in ligand binding.

### 3.9. Dynamic Residue Network Analysis

Dynamic residue network analysis (DRN) was performed on ligand-free and ligand-bound PfHsp70-x and PfHsp70-1 trajectories to identify residues crucial for intraprotein communication, and to further establish related adjustments in the interaction network layout because of ligand binding. Using MD-TASK tools [74], DRN analysis was carried out on 7500 snapshots collected at 10 ps intervals over the last 15 ns of trajectories. Residue interaction network (RIN) graphs were built using *Calculate_network.py* utility, which implements NetworkX libraries. Each snapshot is expressed in terms of nodes and edges: Cβ (Cα for Glycine) atoms represent nodes, and nodes approaching a distance of ≤6.7 Å are adjudged to have formed an edge. Using calc-L and calc-BC options, residue interaction network graphs were analyzed in the context of average shortest path (*L*) and betweenness centrality (*BC*) respectively. Average shortest path is a pointer of residue accessibility. Considering a protein having *t* total residues, the average shortest path value of a residue *P* is the mean distance of shortest pairwise distances between *P* and every other residue (*P*_1_, *P*_2_, … *P*_t_). Betweenness centrality describes the degree of connectivity of a residue *P* by considering the frequency of its involvement in the shortest pairwise paths of every other residue. DRN was computed from the block average of RIN graphs using *avg_network.py* module, hence average *L* and average *BC*. In order to make comparisons between RMSF, average *L* and average *BC*, values of respective metrics were normalized as described before [43].

### 3.10. Coarse-Grained Dynamic Residue Network Analysis

Coarse-grained dynamic residue network analysis (CG-DRN) was similarly performed on ligand-free and ligand-bound PfHsp70-x and PfHsp70-1 trajectories to determine if CG simulations combined with DRN analysis could identify communication networks and recapitulate results of all-atom simulations. For CG-DRN analysis, a graph-based representation of protein structures is employed in which residues are treated as network nodes and inter-residue edges represent residue interactions [73,91,92]. The new version of NAPS approach [93,94] was used for construction of the residue interaction networks and subsequent residue-based network centrality analysis. For our analysis, an interaction strength-based graph representation of protein structures was used in which a residue is considered as a node in the network and an edge is constructed if the interaction strength between two residues is more than the threshold of 4%. The pair of residues with the interaction $I_{ij}$ greater than a user-defined cut-off ($I_{min}$) are connected by edges and produce a protein structure network graph for a given interaction cutoff $I_{min}$. The interaction strength $I_{ij}$ is considered as edge weight. The edges in the residue interaction networks were weighted based on the defined interaction strength and dynamic residue correlations couplings [73,91]. We employed the new option of the updated NAPS package to analyze trajectories. By using 1000 frames of CABS-CG trajectories, NAPS computed the average single network representing the ensemble of simulation trajectories. The average network was constructed using Cα representation of trajectories. In this case, Cα of amino acid residue is considered as node and an edge is drawn if two nodes share an edge for 60% of the simulation. Using the constructed protein structure networks, the residue-based *BC* parameters were computed with the new NAPS server [94].

## 4. Conclusions

In this study, 623 South African natural compounds were docked against potential allosteric regions located in the SBD of PfHsp70-x, PfHsp70-1 and Hsc70 with the aim of identifying selective allosteric modulators. As the identification of such inhibitors, especially against evolutionary conserved targets, presents a challenge, potential inhibitors were chosen not only according to binding energy differences between plasmodial and human protein-ligand complexes but also shape complementarity to the ligand. 

Five compounds were, then, taken into MD simulations. Two sets of experiments were studied: Set1 involving protein models less endogenous modulators (PfHsp70-x Apo, PfHsp70-x-ligand complex, PfHsp70-1 Apo, and PfHsp70-1-ligand complex) and Set2 including protein models consisting endogenous modulators (PfHsp70-x endo-apo, PfHsp70-x endo-complex, PfHsp70-1 endo-apo, PfHsp70-1-endo-complex). Out of five, two ligands, SANC190 and SANC651, showed considerable modulation effects from all-atom MD simulations. SANC190 and SANC651 displayed considerable allosteric regulation characteristics. SANC190 (Millecrone A) is found in extracts of the nudibranch *Leminda millecra* [63], and SANC651 (Warburganal) is extracted from the stem bark of *Warburgia salutaris* [64]. SANC651 possesses potent antifungal and antibacterial activities [64,65]. 

We further compared the dynamic profiles for studied systems obtained from all-atom and coarse-grained simulations. CABS-CG simulations of the studied systems revealed very similar general trends. Overall, we observed that binding of SANC190 and SANC651 ligands can affect conformational dynamics of PfHsp70-x and PfHsp70-1 proteins. Taken together the results and analysis of all-atom and CG simulations, it was evident that SANC190 and SANC651 strongly modulate conformational dynamics of PfHsp70-x and PfHsp70-1 and could consequently regulate succeeding ADP/substrate release events. While both ligands induced substantial structural re-adjustments favoring formation of an NBD-SBD interface in PfHp70-x (considering Set1 results), structural rigidity was conferred to a more dynamic PfHsp70-1. Based on DRN analysis of PfHsp70-x, ligand binding and consequent interdomain advancements majorly influenced the betweenness values of critical residues previously implicated in ATP-induced closed to open conformation transitions, hinting a potential allosteric agonism impact. These findings were also supported by CG simulations, offering further support to our conclusions.

Further, previously, we established protocols to identify allosteric sites by combining MD, PRS and DRN, and to identify allosteric modulators via docking, MD and DRN analysis [11,43]. This study demonstrated that the process of identification of allosteric modulators might be hastened by coarse-grained version of the MD and DRN calculations. 

## Figures and Tables

**Table 1:** Tabulated summary of best ranked docking solutions. All compounds were docked to the β-SBD back pocket in both PfHsp70-x and PfHsp70-1.

Compound	Name	Chemical Formula	PfHsp70-x		PfHsp70-1	
			Cluster Size	Average Binding Energy (kcal/mol)	Cluster Size	Average Binding Energy (kcal/mol)
SANC190	Millecrone A	C_15_H_24_O	75	−7.13	65	−7.03
SANC451	Varacin	C_10_H_13_NO_2_S_5_	45	−8.68	39	−8.73
SANC636	Montabuphine	C_17_H_19_NO_4_	83	−8.82	58	−8.26
SANC649	Isopolygodial	C_15_H_22_0_2_	73	−7.23	31	−7.16
SANC651	Warburganal	C_15_H_22_0_3_	68	−7.47	26	−7.70

## Supplementary Materials

Supplementary materials can be found at https://www.mdpi.com/1422-0067/20/22/5574/s1.
