# Supplementary material for: Establishing Computational Approaches Towards Identifying Malarial Allosteric Modulators: A Case Study of Plasmodium falciparum Hsp70s
Source: Int J Mol Sci. 2019 Nov 8;20(22):5574. doi: 10.3390/ijms20225574 (PMC6887781; doi:10.3390/ijms20225574)
Supplement: Supplementary file 1 [file ijms-20-05574-s001.zip › Supplementary_Data/IJMS_supplementary_revised.pdf]

Supplementary Material

Establishing computational approaches towards identifying malarial allosteric modulators: A case study of *Plasmodium falciparum* Hsp70s

Arnold Amusengeri [1], Lindy Astl [2], Kevin Lobb [1,3], Gennady M. Verkhivker [2,4,5] and Özlem Tastan Bishop [1,*]

[1] Research Unit in Bioinformatics (RUBi), Department of Biochemistry and Microbiology, Rhodes University, Grahamstown, 6140, South Africa
[2] Graduate Program in Computational and Data Sciences, Schmid College of Science and Technology, Chapman University, Orange, CA 92866, United States of America
[3] Department of Chemistry, Rhodes University, Grahamstown, 6140, South Africa
[4] Department of Biomedical and Pharmaceutical Sciences, Chapman University School of Pharmacy, Irvine, CA 92618, United States of America
[5] Department of Pharmacology, Skaggs School of Pharmacy and Pharmaceutical Sciences, University of California San Diego, 9500 Gilman Drive, La Jolla, CA 92093, United States of America
* Correspondence: O.TastanBishop@ru.ac.za; Tel.: +27-46-603-8072

Table S1: Sequence retrieval: Tabulated summary of homologous Hsp70 sequences retrieved from NCBI and their respective BLASTP alignment scores.

Sequence ID	Label	Accession No.	R=Reverse BLAST	Total Score	Query Coverage	E-Value	Identity
PfHsp70-1	PfHsp70-1	XP_001349336.1		1066	90%	0.0	82%
			R	1066	98%	0.0	77%
Hsc70	Hsc70	AAK17898.1		939	88%	0.0	72%
			R	942	99%	0.0	70%
P.knowlesi	PKn_N	XP_002258136.1		1073	91%	0.0	82%
			R	1070	91%	0.0	82%
P.maliarae	PMa_N	SBS82885.1		1063	91%	0.0	81%
			R	1060	90%	0.0	81%
P.Ovale	POv_N	SBT32251.1		1060	90%	0.0	81%
			R	1057	91%	0.0	81%
P.vivax	PVi_N	XP0016149721.1		1067	90%	0.0	82%
			R	1064	90%	0.0	81%
P.reichenowi	Pre_N	KYN99383.1		1375	100%	0.0	98%
			R	1375	100%	0.0	98%
P.gaboni	PGa_N	KYO00892.1		1319	100%	0.0	95%
			R	1340	100%	0.0	95%
P.berghei	PBe_N	AAL34314.1		1067	91%	0.0	81%
			R	1063	90%	0.0	81%
H.hammondi	Hha_P	XP_008882675.1		1027	90%	0.0	79%
			R	1024	91%	0.0	79%
N. caninum	NCa_	XP_003883640.1		1027	90%	0.0	79%
			R	1023	91%	0.0	79%
T. gondii	TGo_P	AAC72001.1		1024	89%	0.0	79%
			R	1024	91%	0.0	79%
E.maxima	Ema_P	XP_013334490.1		1019	89%	0.0	78%
			R	1025	99%	0.0	76%
E.necatrix	ENe_P	XP_013439452.1		1017	89%	0.0	78%
			R	1026	94%	0.0	78%
C.cayetanensis	CCa_P	AAO66452.5		1014	90%	0.0	77%
			R	1014	98%	0.0	77%
M. daphnieae	MDa_P	XP_013237595.1		885	94%	0.0	66%
			R	891	99%	0.0	66%
D.discoedium	DDi_N	XP_646617.1		924	89%	0.0	72%
			R	922	95%	0.0	72%
A.subglobosum	ASu_N	XP_012749557.1		914	89%	0.0	72%
			R	913	94%	0.0	72%
D.purpureum	DPu_P	XP_003291509.1		914	89%	0.0	73%
			R	914	95%	0.0	73%
D.fasciculatum	DFa_P	XP_004350535.1		913	89%	0.0	72%
			R	910	95%	0.0	72%
D.lacteum	DLa_P	KYR00848.1		910	89%	0.0	72%
			R	911	94%	0.0	72%
M.crassus	MCr_P	CAC69880.1		929	89%	0.0	73%
			R	927	92%	0.0	73%

S.histriomuscorum	SHi_P	AED86994.1		928	90%	0.0	73%
			R	926	93%	0.0	73%
E.eurystomus	EEu_p	AAA99874.1		924	90%	0.0	73%
			R	924	95%	0.0	73%
S.lemnae	SLe_P	AED86993.1		923	89%	0.0	73%
			R	922	93%	0.0	72%
E.forcardii	EFo_P	AAP51165.1		923	91%	0.0	72%
			R	921	93%	0.0	72%
S.nova	SNo_P	AAB04940.1		919	89%	0.0	73%
			R	920	94%	0.0	73%
E.nobilii	Eno_P	ABI23727.1		916	89%	0.0	72%
			R	914	93%	0.0	72%
P.marinus	PMa_E	XP_002780413.1		1011	94%	0.0	75%
			R	1011	99%	0.0	75%
V.brassicaformis	VBr_E	CEM31018.1		1004	89%	0.0	78%
			R	1013	99%	0.0	75%
C.cohnii	CCo_E	AAM02973.2		974	89%	0.0	75%
			R	976	99%	0.0	72%
S.invicta	SIn_E	XP_011173246.1		973	94%	0.0	72%
			R	973	99%	0.0	72%
C.biroi	Cbi_E	XP_011348101.1		970	89%	0.0	75%
			R	972	99%	0.0	72%
P.minimum	PMi_E	AEV66161.1		968	89%	0.0	74%
			R	970	99%	0.0	72%
V.emeryi	VEm_E	XP_011862691.1		967	89%	0.0	75%
			R	972	99%	0.0	72%
P.dominula	PDo_E	XP_015174687.1		967	89%	0.0	75%
			R	970	94%	0.0	75%
A.echinatior	AEc_E	XP_011059681.1		964	89%	0.0	75%
			R	967	99%	0.0	72%
A.mellifera	AMe_E	NP_001153544.1		963	90%	0.0	75%
			R	964	99%	0.0	72%
T.zeteki	TZe_E	KYQ52133.1		962	89%	0.0	75%
			R	969	99%	0.0	72%
L.humile	LHu_E	XP_012228066.1		961	89%	0.0	75%
			R	966	99%	0.0	72%
Loa loa	LLo_E	XP_003138751.1		961	95%	0.0	71%
			R	961	99%	0.0	72%
C.costatus	CCo_E	KYN05451.1		959	89%	0.0	75%
			R	963	99%	0.0	72%
C.elegans	CEl_E	NP_503068.1		952	89%	0.0	74%
			R	959	99%	0.0	72%
P.tepidariorum	Pte_E	XP_015908911.1		950	89%	0.0	74%
			R	952	99%	0.0	71%
F.chinensis	FCh_E	AAW71958.1		950	89%	0.0	74%
			R	955	99%	0.0	71%
T.Cornetzi	TCo_E	KYN15760.1		964	89%	0.0	75%
			R	966	99%	0.0	72%

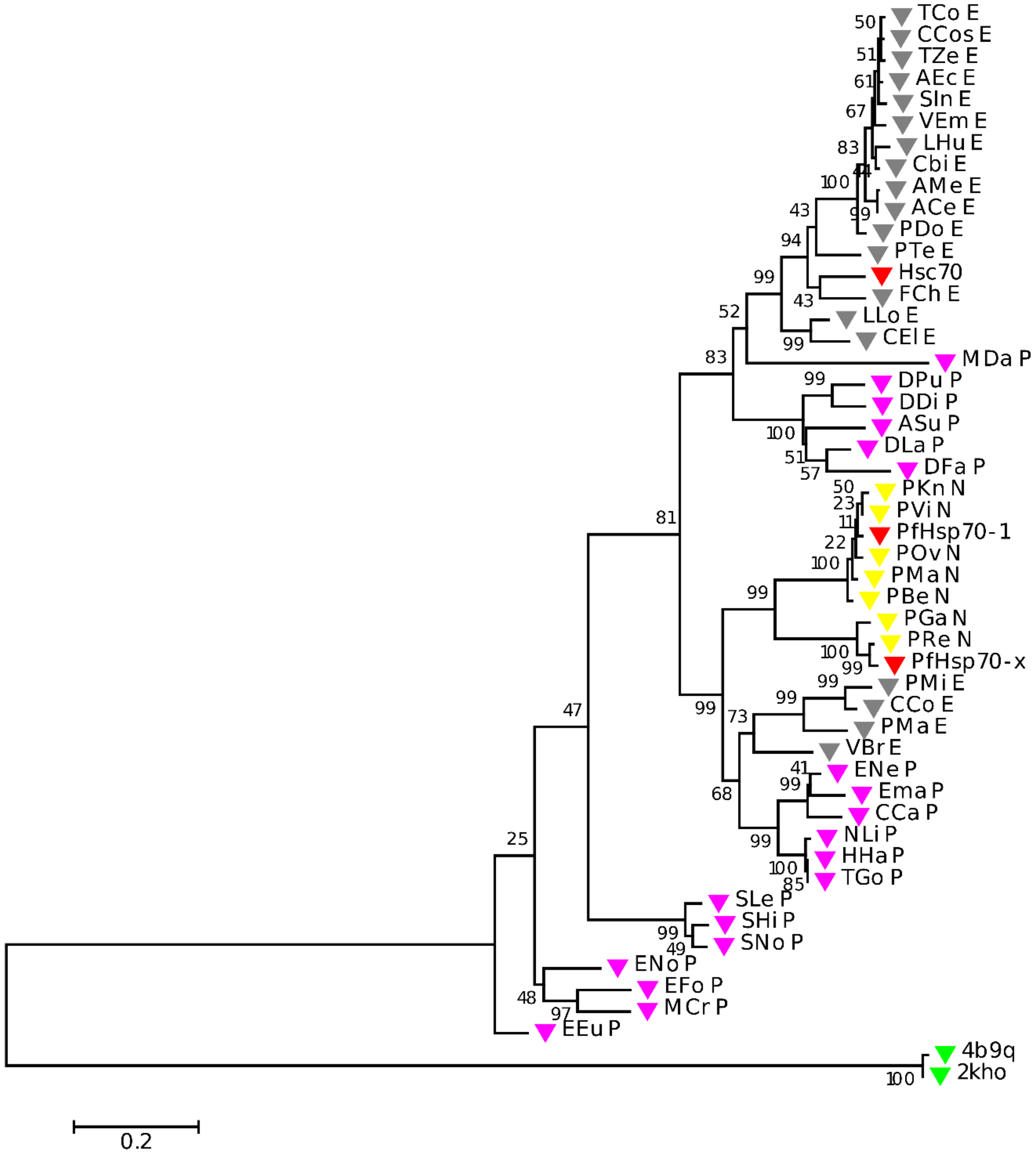

Figure S1: Classification of Hsp70 proteins using MEGA6: Rectangular representation of the phylogenetic tree constructed using Le and Gascuel: Gamma distributed (LG+G) substitution model. 1000 bootstrap replications were performed. Target sequences (PfHsp70-1, PfHsp70-x and Hsc70) are indicated in red. The scale bar represents 0.2 amino acid substitutions per site.

Table S2: Template identification and structure validation results: (**A**) Tabulated summary of template-target sequence identity, query coverage, and template resolution values obtained using PRIMO web server. (**B**) Tabulated results of z-DOPE, PROCHECK Ramachandran plot, and VERIFY3D structure validation tools.

(A)

**Template Identification results**				
Protein	Conformation	Identity	Coverage	Resolution
PfHsp70-x	4b9q (Open)	50%	85%	2.40
	2kho (Closed)	49%	88%	-
PfHsp70-1	4b9q (Open)	48%	88%	2.40
	2kho (Closed)	48%	89%	-
Hsc70	4b9q (Open)	51%	85%	2.40
	2kho (Closed)	52%	85%	-

(B)

**Structure validation results**								
Protein	Conformation	Z-Dope score	PROCHECK (Ramachandran Plot)				VERIFY3D	
			Number of residues in favoured region (Percentage value)	Number of residues in additional allowed region (Percentage value)	Number of residues in generously allowed region (Percentage value)	Number of residues in disallowed region (Percentag e value)	Score	Comment
PfHsp70-x	4b9q (Open)	-1.20	95.20%	4.40%	0.20%	0.20%	87.19%	Pass
	2kho (Closed)	-0.59	93.00%	5.90%	0.70%	0.40%	90.63%	Pass
PfHsp70-1	4b9q (Open)	-1.10	93.80%	4.70%	1.10%	0.40%	91.28%	Pass
	2kho (Closed)	-0.51	92.90%	5.50%	1.30%	0.40%	88.32%	Pass
Hsc70	4b9q (Open)	-1.19	94.70%	4.80%	0.50%	0.00%	93.07%	Pass
	2kho (Closed)	-0.56	94.10%	4.80%	0.50%	0.50%	83.99%	Pass

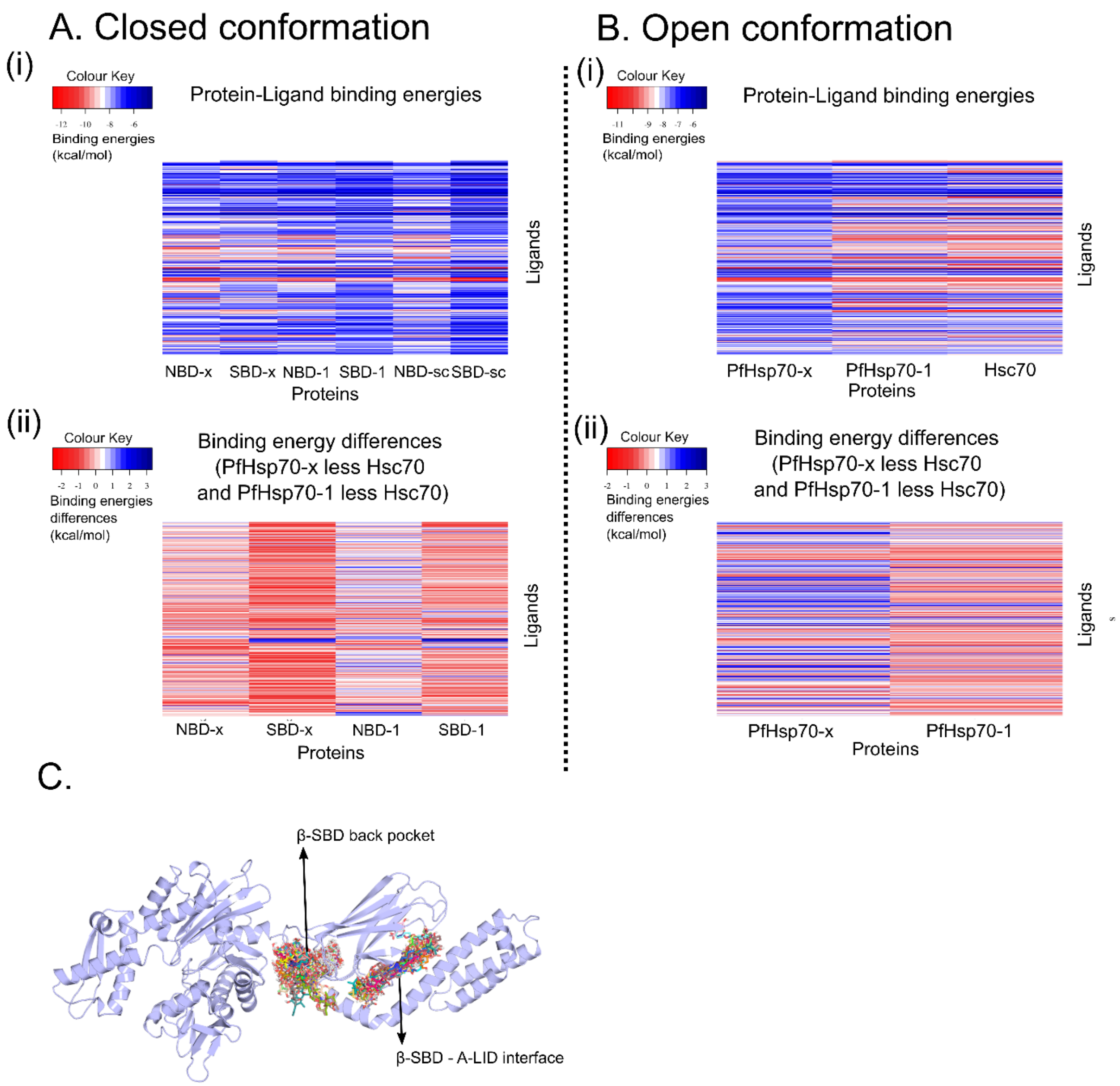

Figure S2: Post-docking analysis: A (i): All closed conformation proteins versus all ligands: Heatmap showing protein-ligand binding energies. **A (ii):** Heatmap showing protein-ligand binding energy differences domain-wise between parasitic (PfHsp70-x, PfHsp70-1) and human (Hsc70) proteins. **B (i):** All open conformation proteins versus all ligands: Heatmap showing protein-ligand binding energies. **B (ii):** Heatmap displaying protein-ligand binding energy differences between parasitic and human proteins. Heatmaps were coloured from red (most negative energy) to blue (most positive energy). **C:** PfHsp70-x model depicting the two distinct ligand-binding sites identified from docking simulations in the substrate binding domain: β-SBD back pocket and β-SBD – A-LID interface.

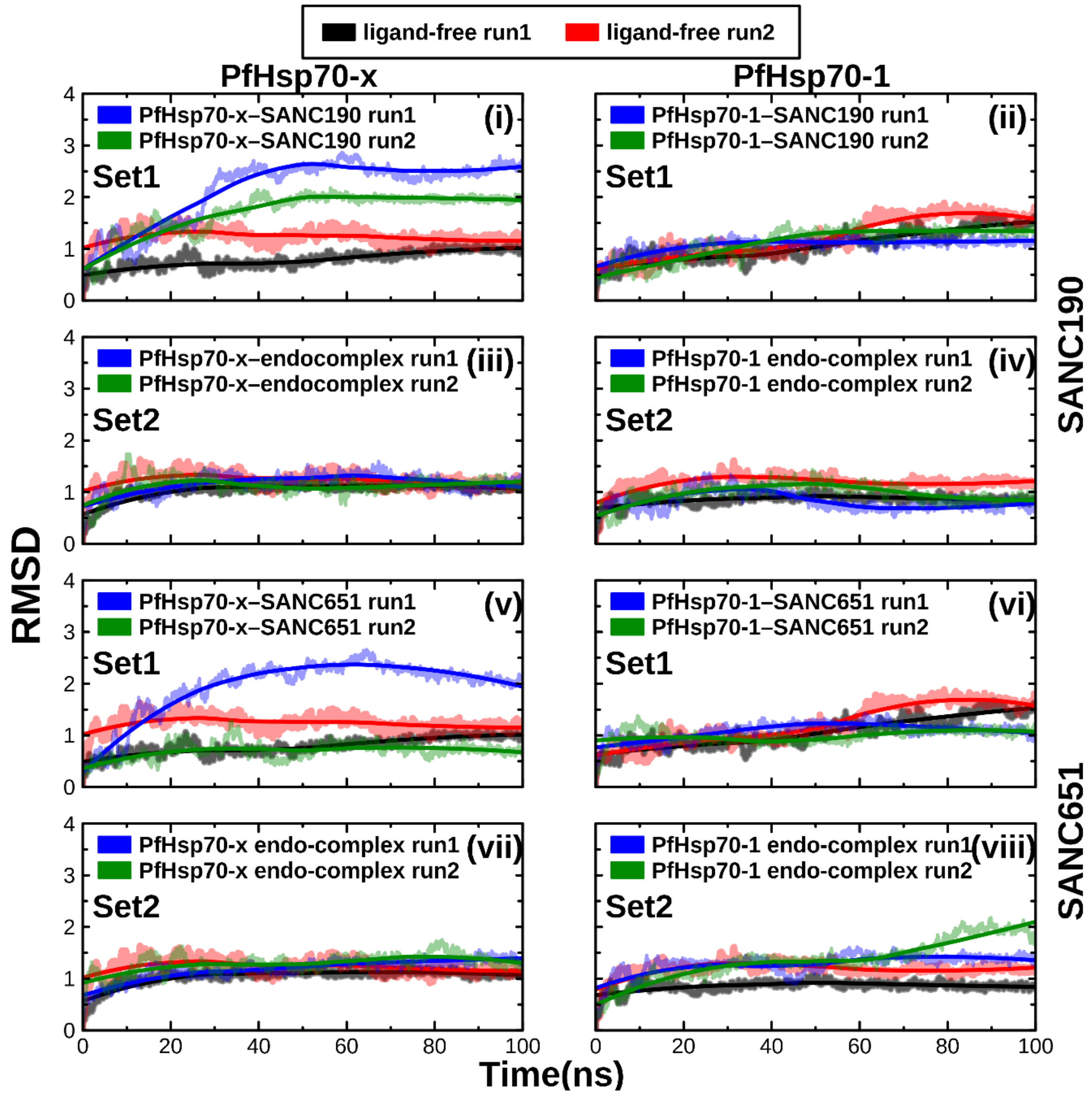

Figure S3: Backbone atoms RMSDs of ligand-bound and ligand-free PfHsp70-x and PfHsp70-1 relative to the starting structure.

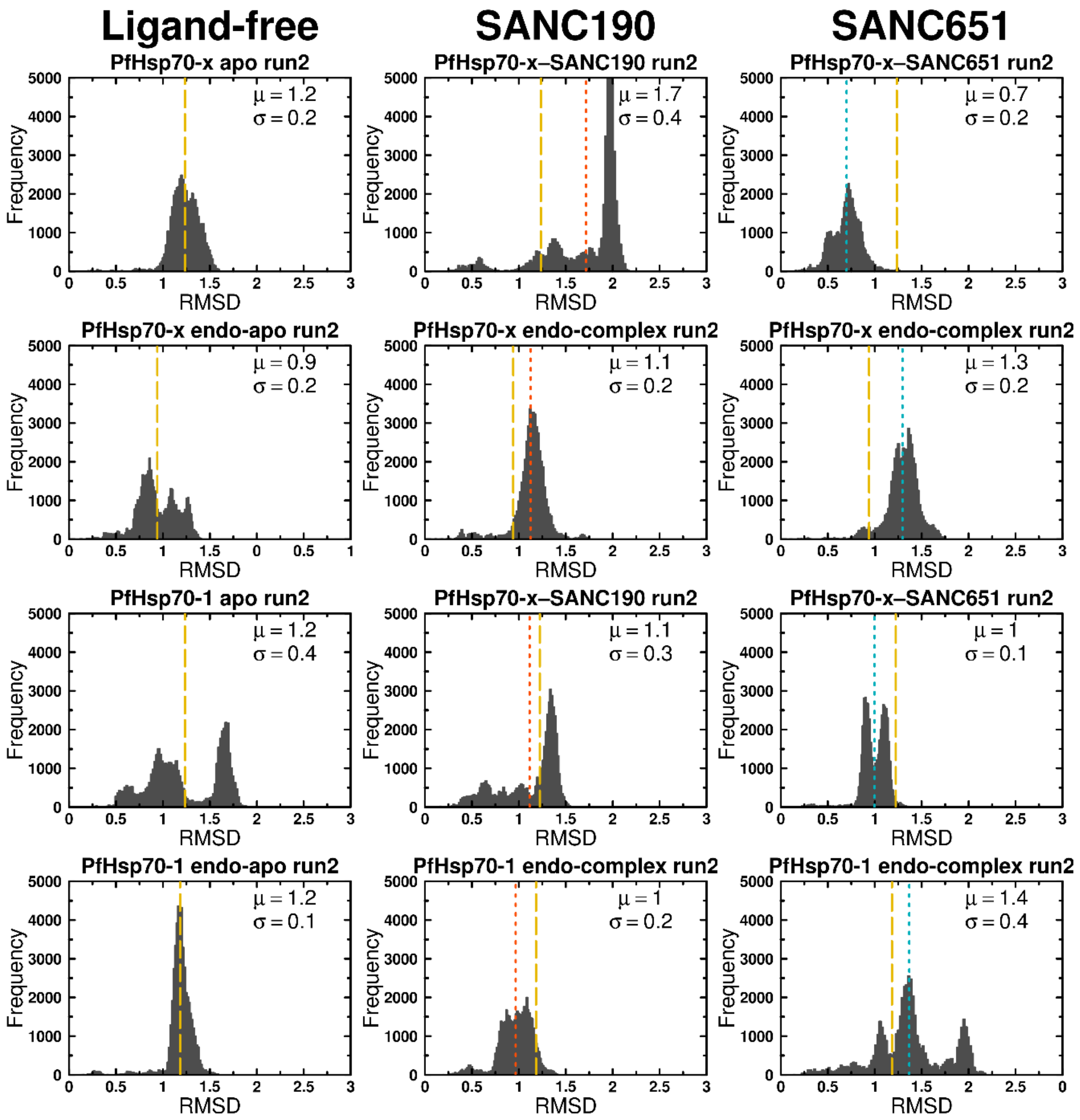

Figure S4: RMSD calculations of duplicate trajectories: Histograms of protein backbone RMSDs versus frequency of occurrence. Dotted lines represent positions of the mean value. Color key: Yellow: ligand-free, Red: SANC190-bound, Blue: SANC651-bound.

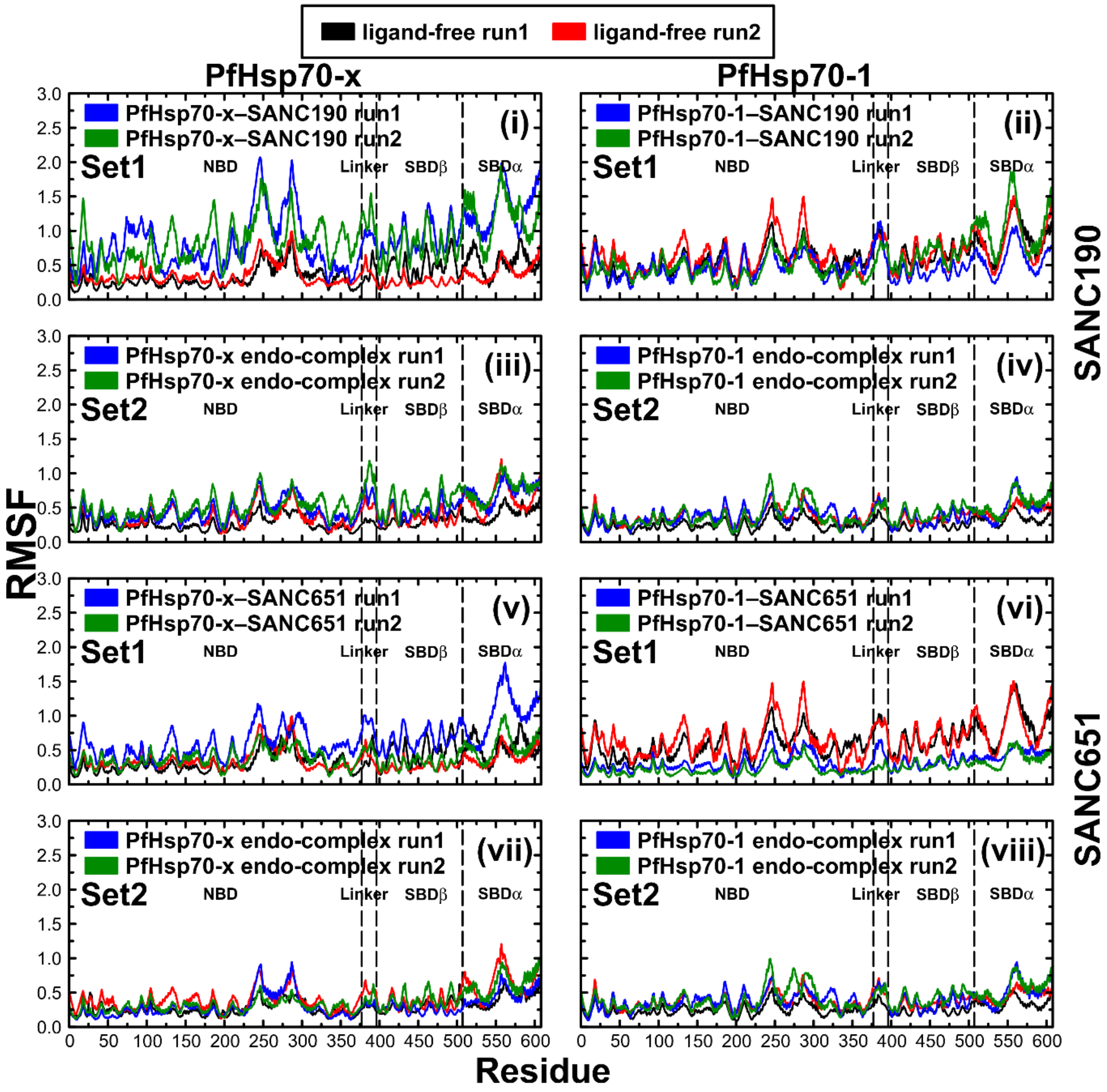

Figure S5: **The root-mean-square fluctuation (RMSF) profiles of PfHsp70-x and PfHSp70-1 computed based on Cα atom positions.**

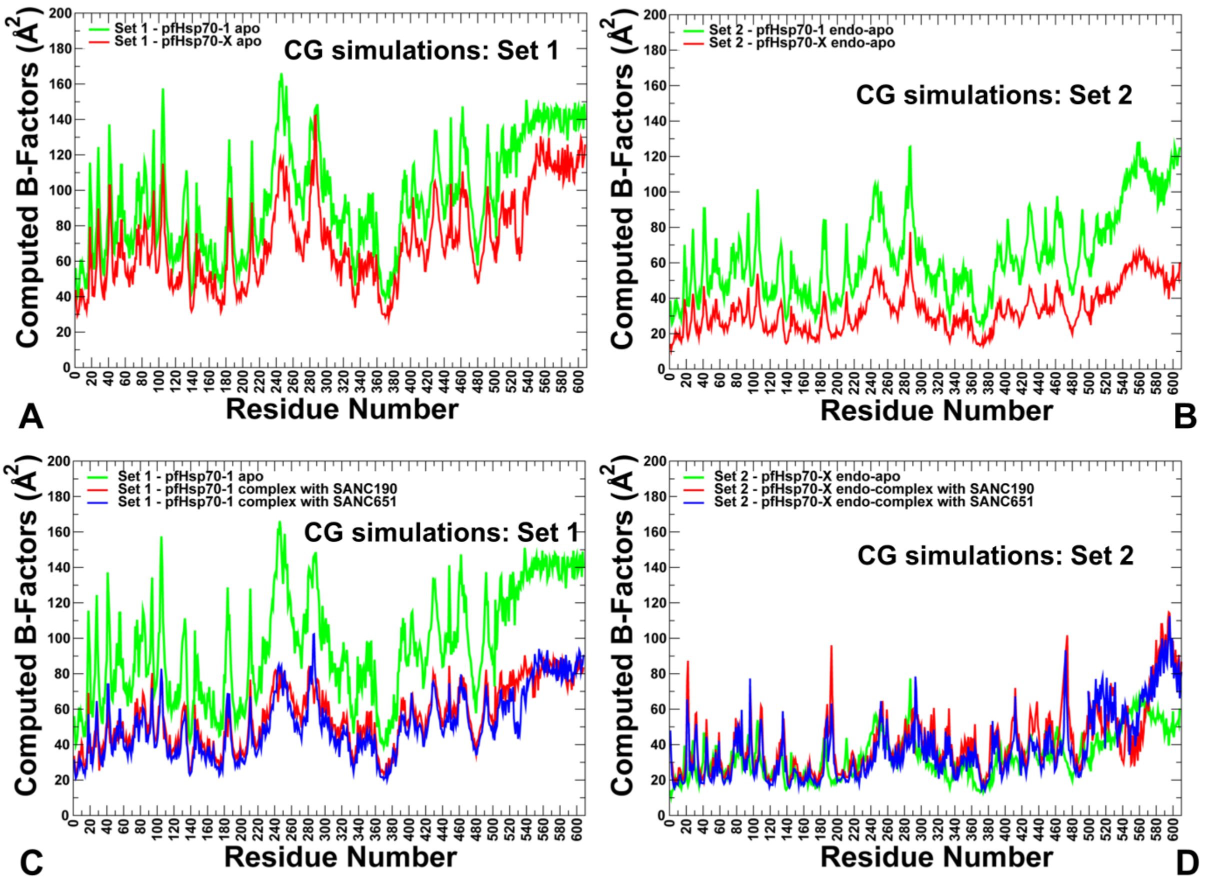

Figure S6: CG conformational dynamics profiles and of the PfHsp70 protein structures (A) The computed B-factors from CG MC dynamics simulations for Set 1 : PfHsp70-1 (apo) in green lines and PfHsp70-x (apo) in red lines. (B) Set 2: PfHsp70-1 (endo-apo) in green lines and PfHsp70-x (endo-apo) in red lines. (C) Set 1: PfHsp70-1 (apo) in green, PfHsp70-1 complex with SANC190 and SANC651 in red and blue lines respectively (D) set 2 : PfHsp70-x (endo-apo) in green, PfHsp70-x complexes with SANC190 and SANC651 in red and blue lines respectively.

Table S3: Principal Component Analysis. Tabulated summary of the top two principal components and the associated percentage variance. Trace values (cumulative sum of eigenvalues) are also shown. (A) PfHsp70-x, (B) PfHsp70-1.

(A)

	Protein systems		Percentage variance accounted by each component		Trace value / cumulative sum of eigenvalues (nm^2)
			PC1 (%)	PC2 (%)	
PfHsp70-x	PfHsp70-x apo	Run1	65.37	12.80	309.46
		Run2	38.03	22.96	256.37
	PfHsp70-x - SANC190 complex	Run1	79.37	9.34	1728.48
		Run2	73.92	14.02	1631.52
	PfHsp70-x - SANC651 complex	Run1	71.41	13.17	942.74
		Run2	56.43	24.96	332.74
	PfHsp70-x endo-apo	Run1	47.57	25.79	176.78
		Run2	66.99	14.44	370.79
	SANC190- bound PfHsp70-x endo-complex	Run1	59.82	23.58	484.48
		Run2	63.65	16.40	653.18
	SANC651- bound PfHsp70-x endo-complex	Run1	68.92	10.17	215.47
		Run2	45.96	26.14	294.07

(B)

	Protein systems		Percentage variance accounted by each component		Trace value / cumulative sum of eigenvalues (nm^2)
			PC1 (%)	PC2 (%)	
PfHsp70-1	PfHsp70-1 apo	Run1	67.24	14.65	821.34
		Run2	77.61	8.51	929.517
	PfHsp70-1 - SANC190 complex	Run1	70.95	15.99	494.78
		Run2	67.82	11.46	786.40
	PfHsp70-1 - SANC651 complex	Run1	50.92	24.20	225.78
		Run2	40.80	16.97	140.50
	PfHsp70-1 endo-apo	Run1	45.90	20.00	140.28
		Run2	39.30	20.60	278.88
	SANC190- bound PfHsp70-1 endo-complex	Run1	57.46	26.43	313.76
		Run2	48.64	22.07	359.72
	SANC651- bound PfHsp70-1 endo-complex	Run1	68.23	14.32	490.66
		Run2	73.42	12.72	477.48

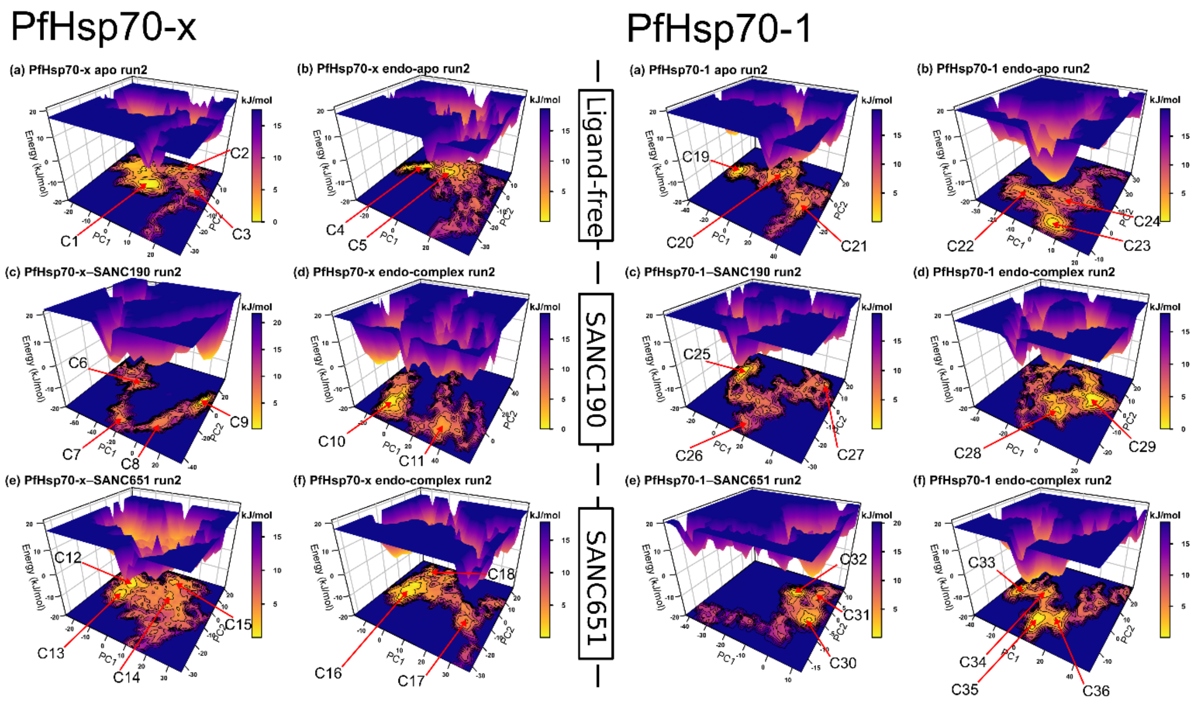

Figure S7: Free energy landscapes of PfHsp70-x and PfHsp70-1 (duplicate runs) computed as a function of PC1 and PC2.

Table S4: Binding free energy values of protein-ligand complexes. The table displays contributions of each energy term to the total binding free energy. Calculations were carried out using the MMPBSA approach.

Protein	Protein-ligand complex		Energy terms				Binding free energy $\Delta G_{binding}$ ($kJmol^{-1}$)
			ΔE_{vdW}	ΔE_{elec}	ΔG_{polar}	$\Delta G_{nonpolar}$	
PfHsp70-x	PfHsp70-x - SANC190 complex	Run1	-106.112 ± 0.238	-6.006 ± 0.128	44.659 ± 0.233	-11.585 ± 0.017	-79.050 ± 0.278
		Run2	-111.228 ± 0.196	-2.771 ± 0.061	33.134 ± 0.193	-11.507 ± 0.016	-92.365 ± 0.253
	SANC190-bound PfHsp70-x endo-complex	Run1	-125.920 ± 0.158	1.616 ± 0.040	37.597 ± 0.104	-11.464 ± 0.012	-98.168 ± 0.190
		Run2	-112.054 ± 0.203	-0.157 ± 0.040	33.234 ± 0.223	-10.861 ± 0.015	-89.817 ± 0.321
	PfHsp70-x - SANC651 complex	Run1	-134.856 ± 0.205	-14.755 ± 0.171	70.573 ± 0.343	-12.617 ± 0.014	-91.645 ± 0.335
		Run2	-106.137 ± 0.238	-17.585 ± 0.164	60.586 ± 0.480	-11.723 ± 0.018	-74.863 ± 0.450
	SANC651-bound PfHsp70-x endo-complex	Run1	-104.138 ± 0.221	-15.297 ± 0.233	61.269 ± 0.261	-12.551 ± 0.015	-70.696 ± 0.272
		Run2	-108.997 ± 0.234	-3.818 ± 0.137	32.190 ± 0.163	-12.015 ± 0.018	-92.649 ± 0.242
PfHsp70-1	PfHsp70-1 - SANC190 complex	Run1	-98.130 ± 0.207	-0.261 ± 0.084	53.949 ± 0.308	-12.044 ± 0.017	-56.488 ± 0.314
		Run2	-107.476 ± 0.218	0.610 ± 0.112	34.378 ± 0.268	-11.671 ± 0.016	-84.151 ± 0.298
	SANC190-bound PfHsp70-1 endo-complex	Run1	-123.747 ± 0.163	8.184 ± 0.053	21.830 ± 0.216	-11.197 ± 0.012	-104.929 ± 0.241
		Run2	-99.403 ± 0.171	1.096 ± 0.058	29.298 ± 0.191	-11.485 ± 0.016	-80.500 ± 0.229
	PfHsp70-1 - SANC651 complex	Run1	-114.367 ± 0.193	-5.571 ± 0.106	33.399 ± 0.259	-11.350 ± 0.014	-97.887 ± 0.274
		Run2	-105.896 ± 0.270	-7.755 ± 0.120	40.257 ± 0.241	-12.088 ± 0.013	-85.473 ± 0.258
	SANC651-bound PfHsp70-1 endo-complex	Run1	-121.057 ± 0.234	-6.095 ± 0.130	55.903 ± 0.182	-12.395 ± 0.015	-83.658 ± 0.288
		Run2	-86.751 ± 1.341	-1.270 ± 0.096	32.708 ± 0.945	-8.557 ± 0.132	-63.977 ± 1.386

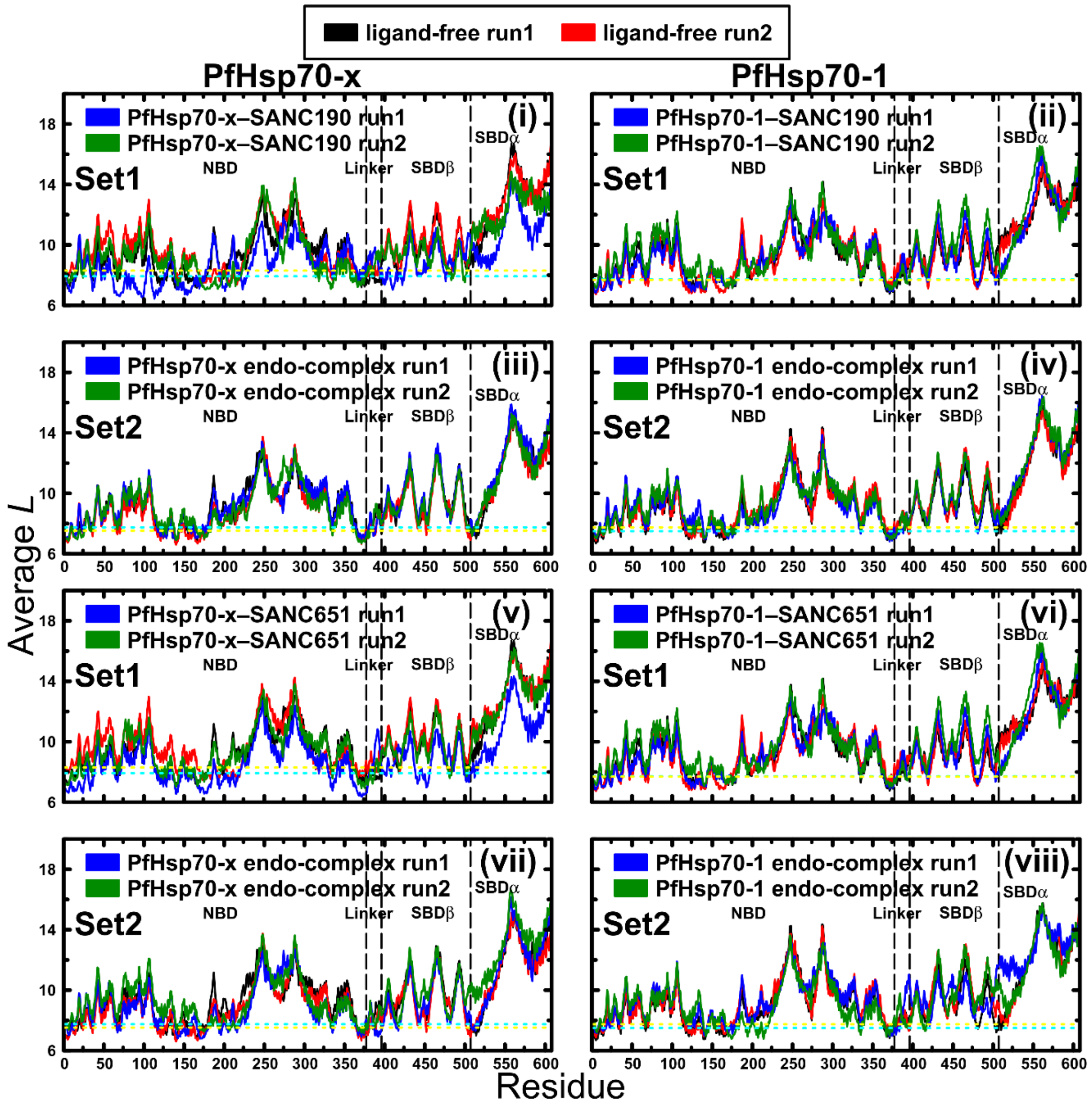

Figure S8: Per residue Average *L* plots comparing ligand-free and ligand-bound systems.

Table S5: Dynamic residue network analysis: Tabulated threshold values applied in identifying low average *L* regions.

Protein	System	Threshold values	
		Average *L* (One standard deviation from mean)	Average *BC* (Two standard deviation from mean)
PfHsp70-x	PfHsp70-x apo (set1)	8.156	0.088
	PfHsp70-x endo-apo (set2)	7.654	0. 087
	PfHsp70-x - SANC190 complex (set1)	7.841	0. 077
	SANC190-bound PfHsp70-x endo-complex (set2)	7.761	0. 084
	PfHsp70-x - SANC651 complex (set1)	7.690	0. 086
	SANC651-bound PfHsp70-x-endo-complex (set2)	7.922	0. 097
PfHsp70-1	PfHsp70-1 apo (set1)	7.719	0. 098
	PfHsp70-1 endo-apo (set2)	7.633	0. 083
	PfHsp70-1-SANC190 complex (set1)	7.840	0. 080
	SANC190-bound PfHsp70-1 endo-complex (set2)	7.736	0. 084
	PfHsp70-1-SANC651 complex (set1)	7.624	0. 084
	SANC651-bound PfHsp70-1-endo-complex (set2)	7.904	0. 091

A)

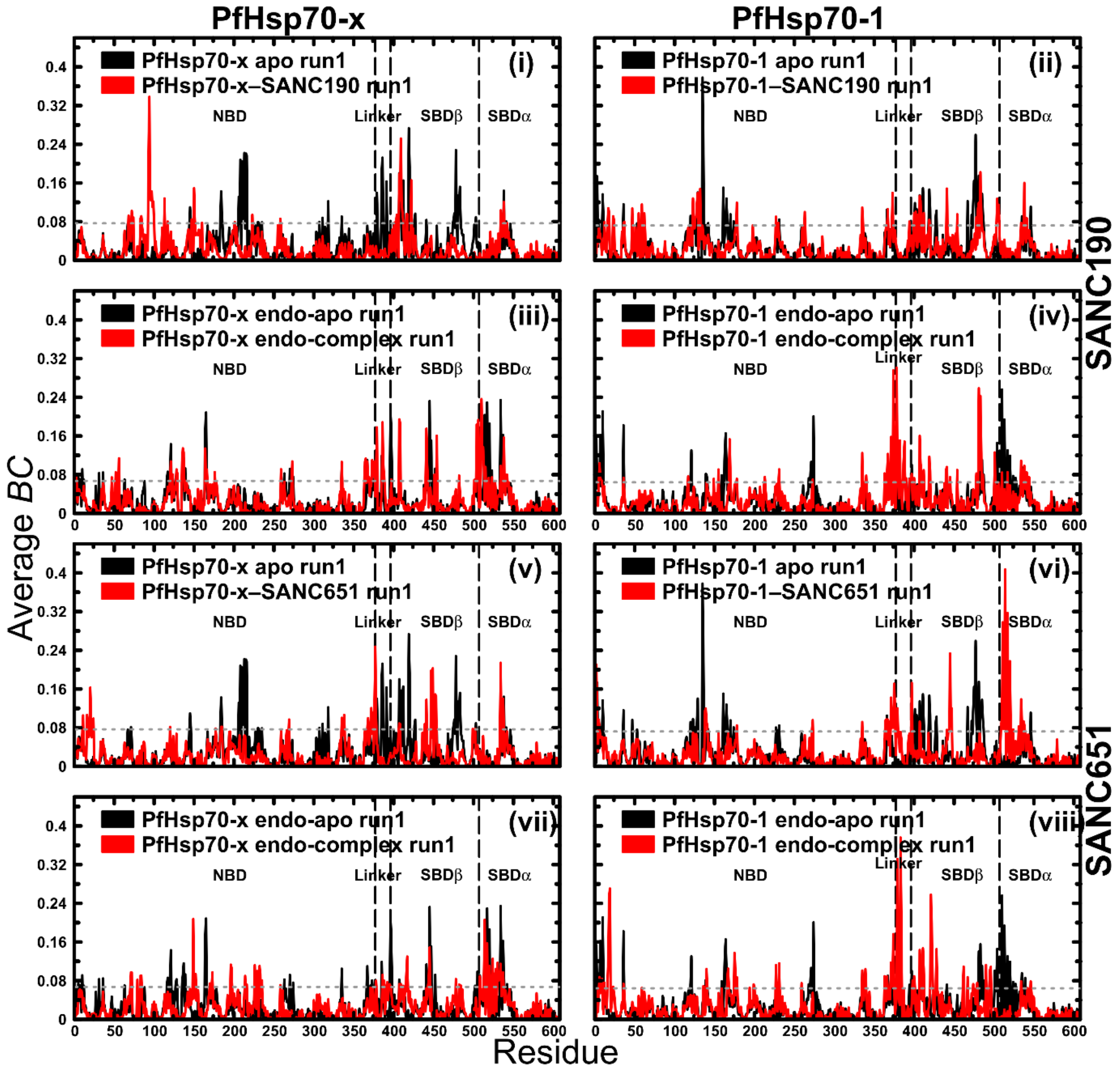
PfHsp70-x
PfHsp70-1
PfHsp70-x apo run1
PfHsp70-x–SANC190 run1
PfHsp70-1 apo run1
PfHsp70-1–SANC190 run1
PfHsp70-x endo-apo run1
PfHsp70-x endo-complex run1
PfHsp70-1 endo-apo run1
PfHsp70-1 endo-complex run1
PfHsp70-x–SANC651 run1
PfHsp70-1–SANC651 run1
(i)
(ii)
(iii)
(iv)
(v)
(vi)
(vii)
(viii)
NBD
Linker
SBDβ
SBDα
SANC190
SANC651
Average BC
Residue
0
0.08
0.16
0.24
0.32
0.4
0
50
100
150
200
250
300
350
400
450
500
550
600

B)

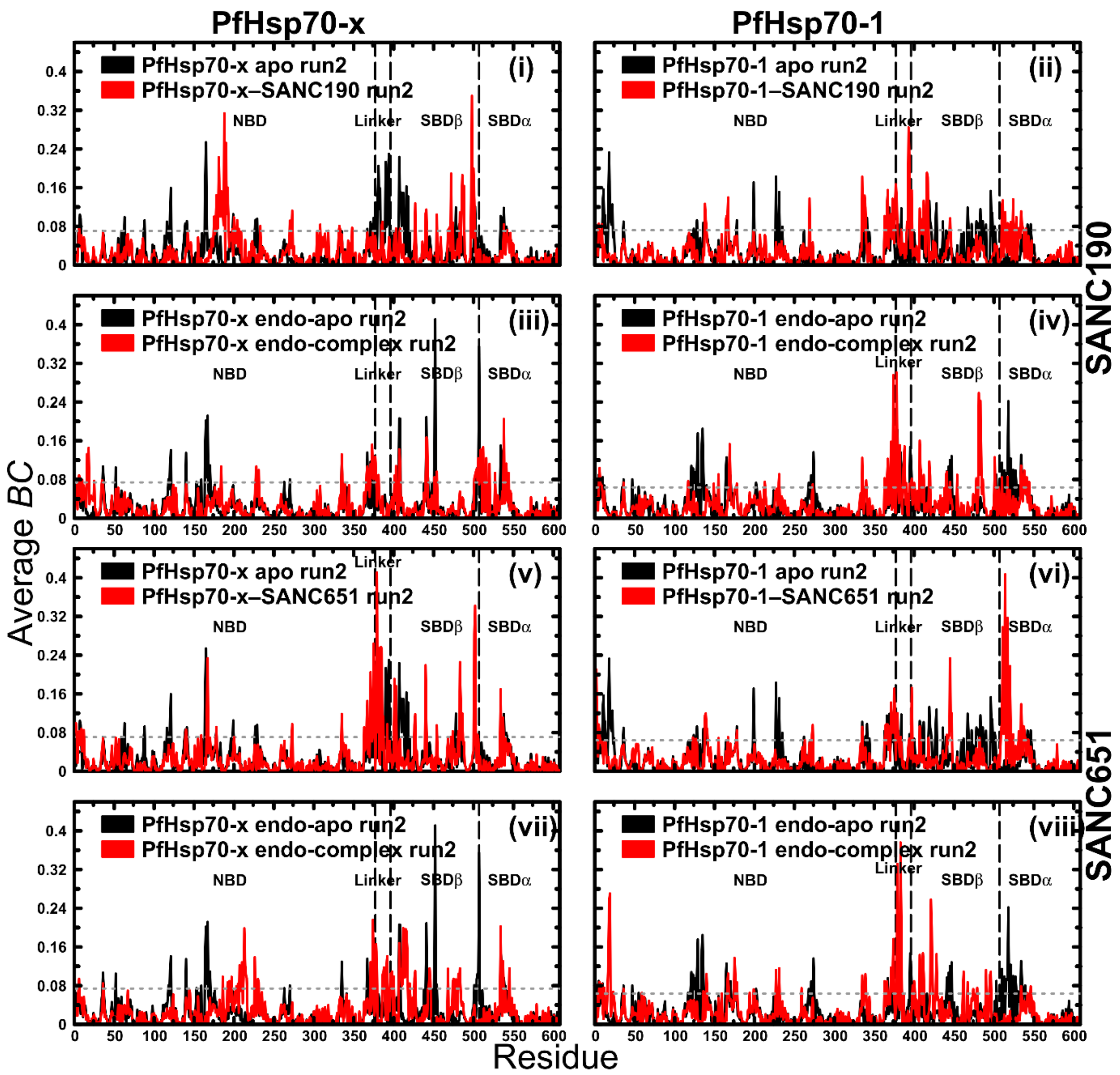

Figure S9: Per residues Average *BC* indices. (**A**): Run 1 trajectories, (**B**): Run 2 trajectories. Colour key: Black: Ligand-free, Red: Ligand-bound.

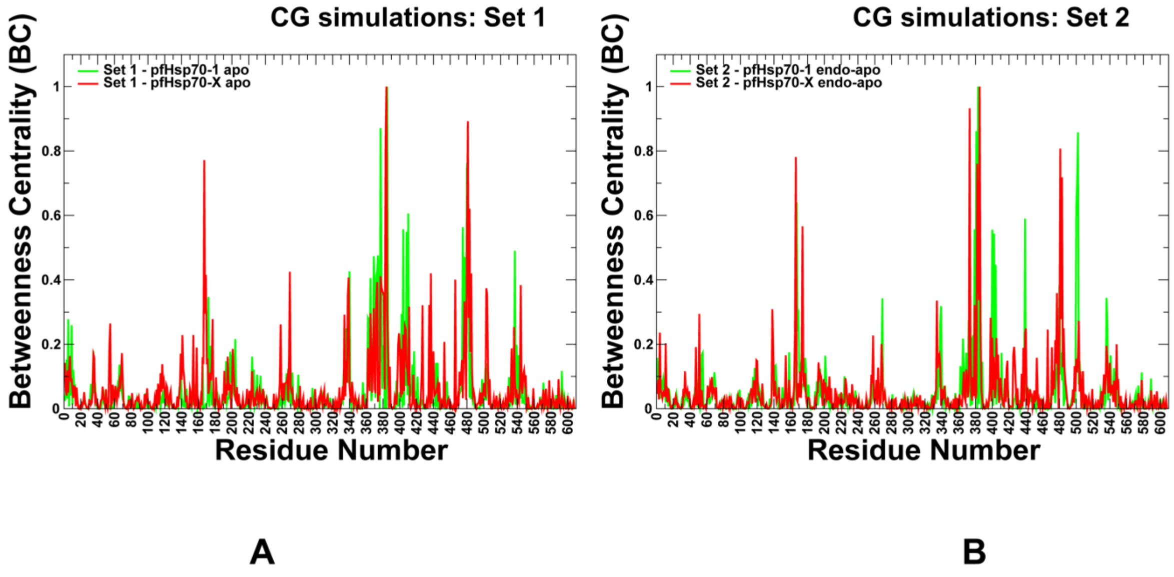

Figure S10: The dynamic network analysis of the PfHsp70 protein structures using CG MC dynamics simulations. (A) The BC profiles for Set 1 : PfHsp70-1 (apo) and PfHsp70-x (apo) in green and red lines respectively. (B) The BC profiles for Set 2: PfHsp70-1 (endo-apo) and PfHsp70-x (endo-apo) in green and red lines respectively.

Table S6: Pearson's correlation coefficient values: Pairwise comparisons of average *BC*, average *L* and RMSF.

**Protein**	**System**	**Run**	***L* vs RMSF (100ns)**	**L^{-1} vs *BC***	**$RMSF^{-1}$ (100ns) vs *BC***
**PfHsp70-x**	PfHsp70-x apo	Run1	0.61	0.28	0.15
		Run2	0.64	0.31	0.23
	PfHsp70-x endo-apo	Run1	0.72	0.48	0.22
		Run2	0.62	0.45	0.11
	PfHsp70-x -SANC190 complex	Run1	0.70	0.55	0.04
		Run2	0.56	0.47	0.15
	SANC190- bound PfHsp70-x endo-complex	Run1	0.57	0.51	0.11
		Run2	0.52	0.52	0.18
	PfHsp70-x -SANC651 complex	Run1	0.74	0.52	0.11
		Run2	0.62	0.45	0.16
	SANC651- bound PfHsp70-x endo-complex	Run1	0.71	0.44	0.03
		Run2	0.81	0.44	0.20
**PfHsp70-1**	PfHsp70-1 apo	Run1	0.58	0.41	0.16
		Run2	0.50	0.45	0.17
	PfHsp70-1 endo-apo	Run1	0.58	0.44	0.16
		Run2	0.52	0.50	0.12
	PfHsp70-1 -SANC190 complex	Run1	0.41	0.50	0.19
		Run2	0.68	0.47	0.12
	SANC190- bound PfHsp70-1 endo-complex	Run1	0.60	0.43	0.22
		Run2	0.60	0.47	0.15
	PfHsp70-1 -SANC651 complex	Run1	0.60	0.48	0.12
		Run2	0.79	0.49	0.20
	SANC651- bound PfHsp70-1 endo-complex	Run1	0.59	0.46	0.03
		Run2	0.71	0.40	0.08

Table S7: Tabulated summary of PfHsp70-x – SANC190 complex and PfHsp70-x – SANC651 complex residues that exhibited substantial changes in average betweenness centrality (*BC*) index. Residues were identified from average values of run1 and run2. Residue numbering corresponds to *E. coli* DnaK sequence (UniProtKB ID: P0A6Y8). Underlined text corresponds to allosteric communication hubs thought to promote opening conformational transitions in *E. coli* Hsp70 from force perturbation experiments [1] .

Protein	Change in average *BC* index	Residues
PfHsp70-x – SANC190 complex	Positive change (Decreased residue centrality)	ASN164, <u>VAL165</u>, LEU208, ASP210, ILE212, PHE213, VAL215, LYS216, <u>ALA375</u>, <u>GLY379</u>, ASP380, GLN381, SER383, VAL385, LEU390, <u>LEU391</u>, ASP392, VAL393, CYS394, <u>MET407</u>, <u>ILE411</u>, <u>GLU412</u>, PRO418, THR419, LYS420, <u>ASP478</u>, GLY481, ILE482, ASN502, ASP503
PfHsp70-x – SANC190 complex	Negative change (Increased residue centrality)	SER93, ASP94, <u>GLY95</u>, <u>LYS96</u>, <u>PRO97</u>, GLN150, LEU181, GLU188, ASN190, GLU472, LEU488, ILE498, <u>ILE500</u>
PfHsp70-x – SANC651 complex	Positive change (Decreased residue centrality)	<u>ASN164</u>, <u>VAL165</u>, LEU208, ASP210, PHE213, VAL215, LYS216, SER383, LYS386, LEU390, <u>LEU391</u>, VAL393, CYS394, <u>MET407</u>, <u>LYS409</u>, <u>ILE411</u>, <u>GLU412</u>, <u>THR416</u>, PRO418, THR419, LYS420, <u>ASP478</u>, ILE482
PfHsp70-x – SANC651 complex	Negative change (Increased residue centrality)	VAL20, ARG167, ALA371, <u>VAL372</u>, ALA374, <u>ALA375</u>, <u>ILE376</u>, LEU377, <u>GLY379</u>, ALA384, <u>GLU401</u>, <u>TYR440</u>, <u>THR447</u>, <u>ASP449</u>, <u>LEU452</u>, <u>LEU453</u>, <u>THR501</u>, <u>ASN502</u>, <u>GLU534</u>, <u>ASN537</u>

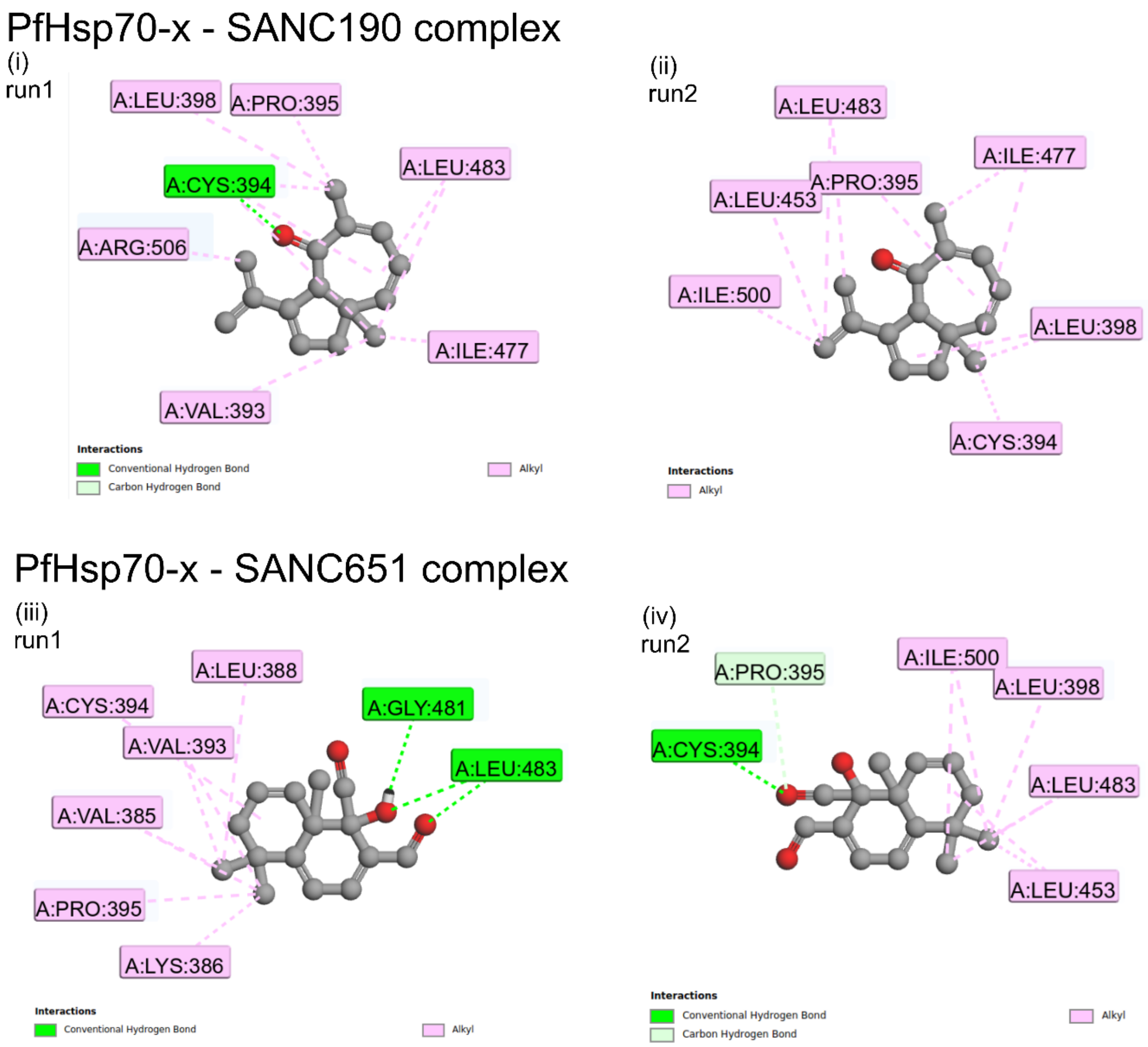

Figure S11: Molecular interactions of PfHsp70-x – SANC190 and PfHsp70-x – SANC651 complexes visualised using Discovery studio.

Table S8: A table summary of residues governing protein-ligand binding affinities. Residues contributing > +/-2.5 kJmol^{-1} of the total binding free energy were identified.

Protein-ligand complex		Per residue contribution to total binding free energy		Standard deviation
		Residue	Binding free energy (kJmol^{-1})	
PfHsp70-x – SANC190 complex	Run1	GLU441<br>VAL393<br>LEU398<br>LEU483<br>ASN502	5.4542<br>-5.6279<br>-3.2236<br>-5.2695<br>-2.8591	0.4711
	Run2	LYS386<br>GLU441<br>LEU398<br>LEU453<br>ILE477<br>ILE482<br>LEU483<br>ASN502	3.6877<br>2.5614<br>-2.9697<br>-2.5790<br>-5.2430<br>-2.8791<br>-6.8433<br>-3.4466	0.5211
PfHsp70-x – SANC651 complex	Run1	LYS386<br>GLU441<br>VAL385<br>VAL393<br>LEU398<br>ILE477<br>ASP478<br>ILE482<br>LEU483	3.2211<br>17.3190<br>-3.6392<br>-3.1065<br>-4.3608<br>-3.8225<br>-2.5636<br>-2.5450<br>-4.8987	0.8722
	Run2	GLU441<br>VAL393<br>LEU398<br>ASP478<br>ASN502	11.2247<br>-4.5537<br>-2.7407<br>-2.7627<br>-2.6827	0.6254

Video S1: All atom MD simulations: An accelerated 100ns video of ligand-free PfHsp70-x (PfHsp70-x apo) .

Video S2: All atom MD simulations: An accelerated 100ns video of PfHsp70-x – SANC190 complex. Colour code: green: protein; purple: SANC190.

Video S3: All atom MD simulations: An accelerated 100ns video of PfHsp70-x – SANC651 complex. Colour code: red: protein; yellow: SANC651.

REFERENCES

1. Penkler, D.; Sensoy, Ö.; Atilgan, C.; Tastan Bishop, Ö. Perturbation-Response Scanning Reveals Key Residues for Allosteric Control in Hsp70. *J. Chem. Inf. Model.* **2017**, *57*, 1359–1374, doi:10.1021/acs.jcim.6b00775.
